# Leaky gut model of the human intestinal mucosa for testing siRNA-based nanomedicine targeting JAK1

**DOI:** 10.1016/j.jconrel.2022.03.037

**Published:** 2022-05

**Authors:** Olga Hartwig, Brigitta Loretz, Adrien Nougarede, Dorothée Jary, Eric Sulpice, Xavier Gidrol, Fabrice Navarro, Claus-Michael Lehr

**Affiliations:** a Helmholtz Institute for Pharmaceutical Research Saarland (HIPS), Helmholtz Centre for Infection Research (HZI), D-66123 Saarbrücken, Germany; b Department of Pharmacy, Saarland University, D-66123 Saarbrücken, Germany; c University Grenoble Alpes, F-38000 Grenoble, France; d CEA LETI, Minatec Campus, F-38054 Grenoble, France; e University Grenoble Alpes, CEA, INSERM, IRIG, Biomics, F-38000 Grenoble, France

**Keywords:** *In vitro* model, IBD, Lipid nanoparticles, siRNA, Tofacitinib, JAK/STAT pathway

## Abstract

Complex *in vitro* models of human immune cells and intestinal mucosa may have a translation-assisting role in the assessment of anti-inflammatory compounds. Chronic inflammation of the gastrointestinal tract is a hallmark of inflammatory bowel diseases (IBD). In both IBD entities, Crohn's disease and ulcerative colitis, impaired immune cell activation and dysfunctional epithelial barrier are the common pathophysiology. Current therapeutic approaches are targeting single immune modulator molecules to stop disease progression and reduce adverse effects. Such molecular targets can be difficult to assess in experimental animal models of colitis, due to the disease complexity and species differences. Previously, a co-culture model based on human epithelial cells and monocytes arranged in a physiological microenvironment was used to mimic inflamed mucosa for toxicological and permeability studies. The leaky gut model described here, a co-culture of Caco-2, THP-1 and MUTZ-3 cells, was used to mimic IBD-related pathophysiology and for combined investigations of permeability and target engagement of two Janus kinase (JAK) inhibitors, tofacitinib (TOFA) and a JAK1-targeting siRNA nanomedicine. The co-culture just before reaching confluency of the epithelium was used to mimic the compromised intestinal barrier. Delivery efficacy and target engagement against JAK1 was quantified *via* downstream analysis of STAT1 protein phosphorylation after IFN-γ stimulation. Compared to a tight barrier, the leaky gut model showed 92 ± 5% confluence, a barrier function below 200 Ω*cm^2^, and enhanced immune response to bacteria-derived lipopolysaccharides. By confocal microscopy we observed an increased accumulation of siJAK1-nanoparticles within the sub-confluent regions leading to uptake into immune cells near the epithelium. A concentration-dependent downregulation of JAK/STAT pathway was observed for siJAK1-nanoparticles (10 ± 12% to 16 ± 12%), whereas TOFA inhibition was 86 ± 2%, compared to untreated cells.

By mimicking the status of severely damaged epithelium, like in IBD, the leaky gut model holds promise as a human *in vitro* system to evaluate the efficacy of anti-inflammatory drugs and nanomedicines.

## Graphical abstract

**Figure:**
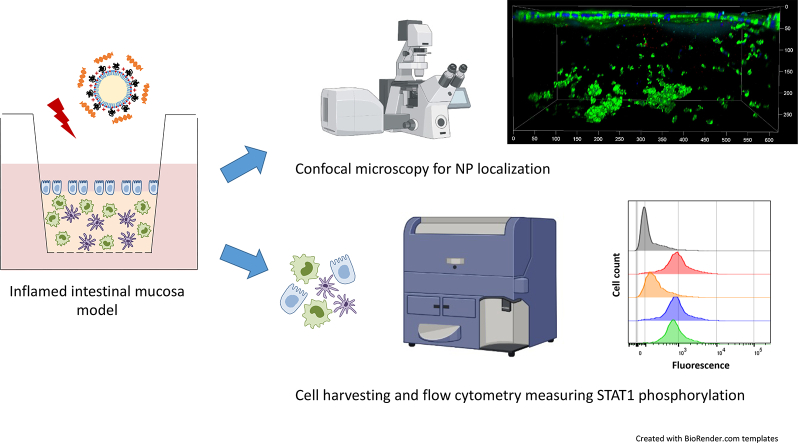

## 1 Introduction

Inflammatory bowel disease (IBD) is characterized by a chronic relapsing inflammation of the gastrointestinal (GI) tract, a common feature in pathologies such as Crohn's disease (CD) and Ulcerative colitis (UC). The etiology of IBD is not fully elucidated, however, genetic and environmental factors are triggering its complex pathogenesis including a dysregulated immune system, dysbiosis of the microbiome and dysfunction of the intestinal epithelial barrier [1]. The translocation of bacterial products into the intestinal wall contributes to an aberrant production of pro-inflammatory mediators (*e.g.* cytokines/chemokines), followed by the infiltration of circulating immune cells and progressive tissue destruction [2]. There is a global evolution of IBD, especially in industrialized countries, with an estimated IBD population of more than ten millions worldwide for 2030 [3].

IBD therapy includes non-specific anti-inflammatory drugs and immunosuppressants or emerging therapeutic strategies with improved specificity such as biologics, however, a substantial percentage of IBD patients suffer from adverse reactions and remain non-responsive or lose responsiveness over time [[4], [5], [6]]. Within the treat-to-target approach to identify novel molecular targets in the treatment of IBD, researchers set the focus on specific signal pathways, such as janus kinase/signal transducer and activator of transcription (JAK/STAT) signal cascade. Shared by multiple cytokines, JAK/STAT is an important therapeutic target in the field of immune-mediated diseases like rheumatoid arthritis (RA), psoriasis and alopecia areata [11]. The only so far approved JAK inhibitor (JAKi) for IBD is tofacitinib (TOFA), a JAK1/3 inhibitor for UC [8]. TOFA entered clinical trials for IBD because of successful use in RA, without animal experimental results, which only were used in retrospective [9]. RA animal models represent the disease more faithfully than so far possible with IBD experimental animal models [10]. Therefore, most immunomodulators investigated in IBD are repurposed from other inflammatory diseases and fail to a significant percentage in IBD [11].

IBD is a multifactorial disease and its pathology divergent. No single model can capture all aspects of disease complexity. Experimental animal models of IBD are *per se* complex model representing a more or less chronic inflammation in dependence of the model selection. Numerous IBD mouse models of acute and chronic inflammation exist such as chemically-induced (*e.g.* dextran sulphate sodium; DSS) or genetically engineered (*e.g.* IL-10 deficiency) murine models [10]. DSS-induced colitis, although commonly used, may not be representative for the immunohistopathology of human IBD [12]. In contrast, a variety of genetic manipulations in mice supported the understanding of immunopathogenic mechanisms involved in chronic inflammation during IBD [11]. However, the current *in vivo* models failed to fully capture the various aspects of human immunity, as well as the human GI-tract anatomy and microbiota [12,13]. In addition, in the case of sequence-specific active molecules such siRNA, the differences in nucleotide sequence prevent the testing of human-optimized lead candidates in animal models.

*In vitro* cell models are representing an important pre-clinical tool for drug development and serve as complementary system to *in vivo* animal testing. However, investigations within multicellular organisms may result in overseen or unrelated experimental outcomes, whereas the simplified context of *in vitro* models provides the precise assignment of pharmacological effects. Recent advances in complex *in vitro* models are challenged by physiological resemblance focusing for example on intestinal organoids or microfluidic systems with dynamic flow or contractile forces. [14,15]. Nevertheless, increased complexity of these models retains some limitations with impact on drug interactions studies. Besides ethical aspects and limited access to human primary tissue, organoid culture has some drawbacks related to high variability between individual samples, difficulties in dosing/sampling for transport studies, and the lack of immune-related responses mediated by immune cells [16,17]. Microfluidic chips are more expensive and challenging for cell harvesting for further analysis. The combination of a simplified, well-controlled and repeatable setup with a chosen degree of complexity focussing a particular cellular response improves drug development and clinical translation*.* In this perspective, *in vitro* intestinal models for drug interaction studies are ranging from simplified 2D cell culture of the epithelial barrier function (*e.g.* Caco-2 colon carcinoma cells) to more advanced 3D co-cultures with essential features of the biological counterpart such as extracellular matrix (ECM) and human-derived cell types for epithelial/immune cell interplay [18]. These *in vitro* models should ideally capture specific disease conditions to investigate the genuine interplay of drug delivery systems and disease-related circumstances.

In this context, recently established 3D co-culture models composed of either human primary [19] or cell line-derived [20] immune cells (macrophages and dendritic cells) were co-cultured with epithelial cells in a physiologically relevant microenvironment based on Transwell® supports. The effects of intestinal inflammation mediated by pro-inflammatory cytokines (*e.g.* IL-1β) or lipopolysaccharides (LPS) were investigated on barrier function and nanoparticle absorption. Under inflamed conditions with elevated cytokine production (IL-8 & TNF-α) and increased permeability, an increased particle uptake with immune cell co-localization was observed [19]. To improve the reproducibility of the model, primary cells were replaced by immune cell lines without affecting the immune response, however, particle uptake was limited to the epithelial cell layer without reaching the underlying sub-epithelial tissue containing immune cells, even under inflamed conditions [20]. To optimize the inflamed state of the model regarding drug delivery system absorption and translocation across the epithelium with increased immune cell uptake, we hypothesized that including a compromised epithelial barrier would provide *in vivo* IBD resemblance. Due to chronic intestinal inflammation aberrant epithelial damage and apoptosis leads to harmful tissue destruction and partial loss of epithelial barrier integrity driven by tight junctions (TJ) openings and epithelial breaches [21,22].

Under these aspects, we developed a so-called leaky gut model with advanced features to mimic severe IBD conditions *in vitro* including a dysfunctional epithelial barrier and the presence of immunocompetent cells arranged in a physiologically relevant microenvironment. Two different pharmaceutical approaches targeting JAK/STAT pathway were investigated within this essential pathopysiological context. On the one hand, a small molecule with high permeability and broad mechanism of action, and on the other side, an alternative compound with high specificity using small interfering ribonucleic acid (siRNA)-based therapeutic approach. In contrast to pan-JAKi TOFA, siRNA was used to specifically target human JAK1 (siJAK1) *via* RNA interference, a natural cellular process regulating gene expression. For local delivery in IBD context and efficient cellular uptake, siJAK1 duplexes were complexed with a protective drug delivery system using cationic lipid nanoparticles (LNPs) with a diameter of 40 nm by electrostatic interactions to form siRNA-loaded nanoplexes.

In this work, we report a complex *in vitro* model of the human intestinal interface mimicking IBD pathophysiology that allows the combined investigation of intestinal barrier crossing as well as efficacy and target engagement of drug/delivery systems exploiting JAK/STAT pathway inhibition. The leaky model required to establish a protocol to control the confluency, and verification on a sufficient number of immune cells to respond to an inflammation trigger. Further important steps in widening the applicability of the co-culture model are on the one side the improved viability testing and, more important, the method of disintegration into single-cell suspension. STAT phosphorylation status of single cells by flow cytometry analysis could therefore be established as molecular downstream signal of JAK-knockdown, first tested with the clinical used pan-JAK inhibitor TOFA. The model could reveal the superiority in the siRNA delivery efficacy of an optimized transfection protocol using LNPs compared to the transfection reagent Lipofectamine RNAiMAX.

## 2 Material and method

### 2.1 Materials

Human colorectal adenocarcinoma cell line Caco-2 (HTB-37) was obtained from American Type Culture Collection (ATCC, Manassas, USA). THP-1, derived from acute monocytic leukaemia (ACC 16), MUTZ-3 cells, a monocytic cell line with dendritic-like phenotype (ACC 295), and urinary bladder cell line 5637 (ACC 35) were purchased from German Collection of Microorganisms and Cell Cultures GmbH (DSMZ; Braunschweig, Germany). Dulbecco's Modified Eagle Medium (DMEM), Roswell Park Memorial Institute (RPMI), α-Minimum Essential Medium (α-MEM), fetal calf serum (FCS) and non-essential amino acids (NEAA), trypsin-EDTA, sodium bicarbonate (NaHCO_3_), penicillin/streptomycin (Pen/Strep), Alexa Fluor (AF)488-conjugated Phalloidin, Calcein AM, and transfection reagent lipofectamine RNAiMAX (Lipo) were obtained from Invitrogen (Carlsbad, USA). Phorbol 12-myristate 13-acetate (PMA), accutase, human AB serum, lipopolysaccharides (LPS) from *Escherichia coli* O111:B4, bovine serum albumin (BSA), saponin, collagenase type II, and 4′,6-Diamidino-2-Phenylindole Dihydrochloride (DAPI) were purchased from Sigma-Aldrich (Taufkirchen, Germany). Collagen type I solution (PureCol; 3 mg/mL) was obtained from Advanced Biomatrix (Tucson, USA). Transwell® Permeable Supports (#3460; polyester (PET) membrane with 0.4 μm pore size) were purchased from Corning (New York, USA). Paraformaldehyde (PFA) was obtained from Electron Microscopy Sciences (Hatfield, USA). Double-faced adhesive tape was ordered from Coroplast (Wuppertal, Germany). Fluorescence mounting medium was obtained from Agilent Technologies (Santa Clara, USA). CBA Human soluble Protein Flex Set for tumor necrosis factor-alpha (TNF-α) and interleukin-8 (IL-8), AF647 mouse anti-phospho-STAT1 (pY701) antibody and AF647 mouse IgG 2a, *κ* isotype control, Perm Buffer III were purchased from BD Biosciences (Heidelberg, Germany). FITC-conjugated mouse anti-CD14 and CD11b were purchased from Biolegend (San Diego, USA). Recombinant human interferon gamma (IFN-γ) was purchased from Miltenyi Biotec (Bergisch Gladbach, Germany). Tofacitinib citrate (TOFA; CP-690550) was obtained from Selleckchem (Houston, USA).

### 2.2 Cell culture

All cell lines were maintained (up to 20 passages after thawing) at 37 °C and 5% CO_2_ atmosphere and a relative humidity of 95%. Caco-2 cells were cultured in DMEM supplemented with 10% FCS and 1% NEAA. Sub-confluent Caco-2 cultures (70–80%) were passaged once a week using 0.05% trypsin-EDTA with split ratio of 1:20. THP-1 cells were grown in suspensions in RPMI supplemented with 10% FCS and sub-cultured every 3 or 4 days with a split ratio of 1:4 or 1:6, respectively. MUTZ-3 cells were cultured in suspensions in α-MEM supplemented with 20% FCS and 20% conditioned medium and split weekly with a ratio of 1:3. Conditioned medium was prepared by growing the 5637 in RPMI supplemented with 10% FCS and medium collection every second day. Sterile-filtered conditioned medium was kept at −20 °C until further use.

### 2.3 Experimental setup of leaky gut model

**Fig. 1:**
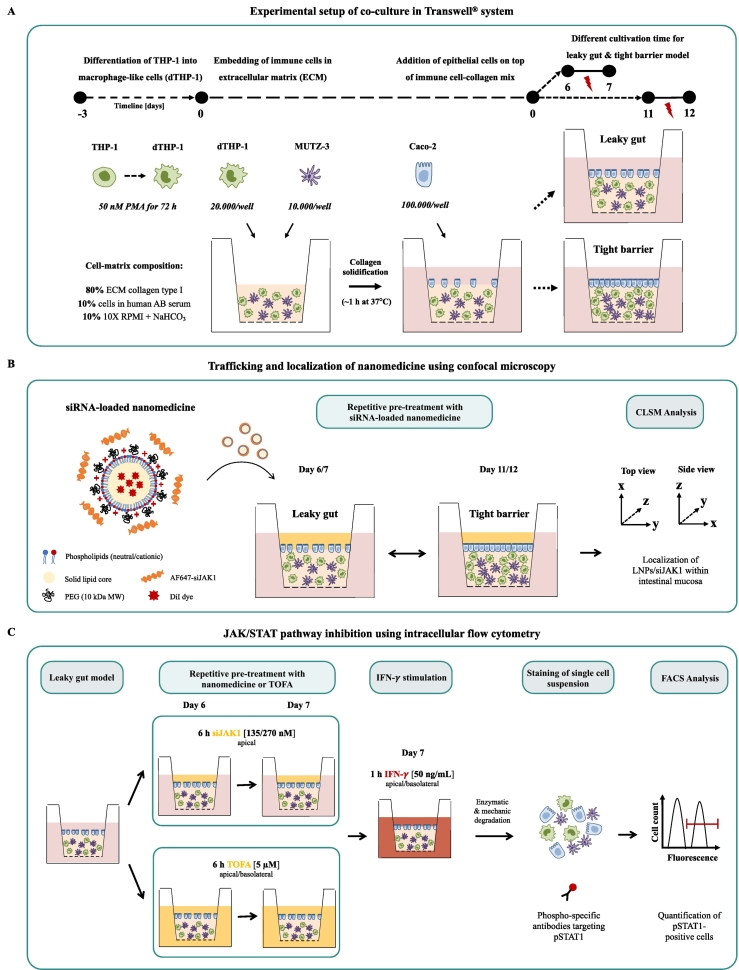
Experimental setup of co-culture model mimicking the human intestinal mucosa (A) and co-culture treatment using siRNA-based nanomedicine for localization studies (B) and JAK/STAT pathway inhibition (C). Co-culture model composed of three different cell types was arranged in a physiologically relevant microenvironment and placed in a Transwell® system. PMA-differentiated THP-1 cells (dTHP-1) were embedded together with dendritic-like cells (MUTZ-3) in a collagen matrix in a ratio of 2:1. After solidification, epithelial cells (Caco-2) were seeded on top and cultivated for 6 days to represent a sub-confluent epithelial barrier (Leaky gut model) instead of previous 11 days comprising a tight epithelium (Tight barrier model). Inflammation of the co-culture was induced by LPS-stimulation (indicated red flash). For localization studies of siRNA-loaded nanomedicine, co-culture was treated with fluorescently-labeled nanocarrier (DiI-LNPs) and siRNA (AF647-siJAK1) and analyzed by confocal microscopy. For JAK/STAT pathway inhibition, co-culture was pre-treated with siJAK1-loaded LNPs or TOFA and subsequently stimulated with IFN-γ. The downregulation of JAK/STAT signaling was evaluated by downstream analysis of STAT1 protein phosphorylation using phospho-specific antibody staining and flow cytometry. (For interpretation of the references to colour in this figure legend, the reader is referred to the web version of this article.)

The starting point of the leaky gut model was a previously published tight barrier model of the human intestinal mucosa to study the effect of acute inflammation [20]. Compared to the previous model, several alterations were introduced to the leaky gut model. The differentiation of THP-1 cells into macrophage-like cells (dTHP-1) was changed using a higher and longer exposure to PMA to generate a more active and differentiated macrophage-like phenotype (surface marker, cell morphology, and cytokine production) [23]. Moreover, we have doubled the number of dTHP-1 cells in the leaky gut model since dTHP-1 cells were identified as the main contributors for cytokine production [19]. Finally, to mimic impaired barrier properties (“leaky gut”) like in chronic inflammation, the model was already used after 6 days of cultivation representing a sub-confluent epithelial cell monolayer compared to previous cultivation period of 11 days with a tight epithelium (“tight barrier”), as illustrated in Fig. 1A.

Before co-culture start, monocytic THP-1 cells were differentiated into macrophage-like cells (dTHP-1) using 50 mM PMA for 72 h and harvested by accutase. A total amount of 20.000 dTHP-1 cells was embedded together with 10.000 MUTZ-3 cells in 80% (*v*/v) collagen type I solution containing 10% 10× RPMI +20 mg/mL NaHCO_3_ and 10% human AB serum. A total volume of 150 μL was placed into the apical chamber of the Transwell® insert. After 1 h  of solidification at 37 °C and 5% CO_2_, Caco-2 cells (100.000/well) were seeded on top of the collagen surface, in a total volume of 0.5 mL of Caco-2 medium containing 1% Pen/Strep. Subsequently, 1.5 mL of THP-1 medium plus 1% Pen/Strep were added to the basolateral side and cells were cultured under submerged conditions with media exchange every 2 to 3 days.

### 2.4 Transepithelial electrical resistance (TEER)

TEER was determined with an Epithelial Volt Ohm Meter (EVOM2; World Precision Instruments, Sarasota, USA) equipped with STX2 chopstick manual electrodes. To minimize the factors affecting TEER, all measurements were performed before medium replenishment at controlled temperature, placing the Transwell® plate on a pre-warmed heating plate at 37 °C. Final TEER values, represented in units of Ω*cm^2^, were calculated by subtracting the blank value (*R*_*blank*_, resistance of the filter support without cells, ~95 Ω) from the sample value (*R*_*total*_) and multiplying it by the effective growth area (*A*) of the Transwell® insert (1.12 cm^2^):

$$\mathrm{TEER}\;\left[\Omega \;x\;{\mathrm{cm}}^{2}\;\right]=\left(R_{\mathrm{total}}-R_{\mathrm{blank}}\right)\;x\;A$$

Co-cultures with TEER values above 300 Ω*cm^2^ were considered having an established epithelial barrier property. For barrier integrity changes upon inflammation with LPS, TEER was measured before and after 6 and 24 h after inflammation induction. TEER values are represented as percentage, normalized to of the initial TEER value (before addition of LPS).

### 2.5 Cytokine measurements after LPS stimulation

The presence of cell-secreted cytokines in co-culture supernatants was determined using BD™ Cytometric Bead Array (CBA; BD Biosciences, Heidelberg, Germany) and a fluorescence activated cell sorter (FACS; LSR II Fortessa, BD Biosciences). Aliquots of cell culture supernatant from both, apical and basolateral compartment of the Transwell® system, were collected before and after 6 and 24 h of inflammation with LPS (10 μg/mL). Experiments were performed in triplicates and supernatants were stored at −80 °C before analysis. Cytokine quantification was performed using CBA Human soluble Protein Flex Set for TNF-α and IL-8 in accordance with the manufacturer's protocol. Analysis was performed using FCAP Array™ Software Version 3.0.1 (BD Biosciences) and final cytokine concentration is represented in units of pg/mL.

### 2.6 Staining procedure, confocal microscopic analysis and surface area measurements for Caco-2 confluence calculation

Co-culture was fixed with 4% (*v*/v) PFA for 30 min at room temperature (RT) and washed twice with PBS. For F-actin staining, cells were first permeabilized with 1% (*w*/*v*) BSA 0.05% (w/v) saponin in PBS for 20 min at RT and afterwards stained with AF488-conjugated Phalloidin for 30 min at RT. Afterwards, cell nuclei were stained with 5 μg/mL DAPI for 30 min at RT. Fluorescence imaging of co-cultures grown in Transwell® systems required the removal of the whole collagen-cell matrix plus supporting PET membrane from the insert. After two washing steps, remaining PBS was removed, and the insert flipped upside down. Using a scalpel, the edges around the PET membrane were carefully cut and the collagen-cell matrix plus membrane transferred to a microscope slide with the cells facing up. After drying, cells were surrounded by a 0.5 μm thick double-faced adhesive tape to prevent deformation of the collagen-cell matrix. Finally, cells were covered with fluorescence mounting medium and sealed with coverslips. Mounted co-culture was stored in the dark at 4 °C until analysis with confocal laser scanning microscopy (CLSM; Leica TCS SP8; Leica, Mannheim, Germany). Fluorescence of DAPI and AF488-phalloidin was detected using HyD detectors with emission filters between 440 - 500 nm (405 nm laser) and 520–550 nm (488 nm laser), respectively. Images were acquired at 1024 × 1024 resolution using a 25× water immersion objective (Fluotar VISIR 25×/0.95). Confocal imaging was performed by collecting images from multiple optical planes in the z-dimension (z-stacks) with a thickness of around 300 μm and 0.57 μm z-step size. Image analysis was performed using software Leica Application Suite X (LAS X; Leica, Mannheim, Germany). Image sequences acquired from z-stack scans were merged to create a 3D reconstruction of the specimen and adjusted in terms of fluorescence intensity, rotation in all dimensions, such as top (xy-) or side (xz/yz-dimension) view of 3D volume projections and exported as tiff files.

The expression of CD14 and CD11b in monocytic (THP-1) and PMA-differentiated cells (dTHP-1) was analyzed by FACS. After harvesting, 1 × 10^6^ cells of (d)THP-1 cells were stained with FITC mouse anti-CD14 or CD11b for 1 h at 4 °C. Cells were washed and resuspended in 2% FCS in PBS prior to FACS analysis of FITC-positive cells. For CLSM, dTHP-1 cells/well (200.000/well; μslide) were fixed with 4% (*v*/v) PFA, blocked with 5% FCS for 30 min at RT and stained with FITC mouse anti-CD14 or AF647 anti-CD11b for 1 h at RT. Afterwards, cells were stained for cytoskeleton (Rhodamine or AF488-phalloidin) and cell nuclei (1 μg/mL DAPI).

Surface area of sub-confluent regions within the leaky gut model was calculated by ImageJ (version 1.52, NIH, Bethesda, USA). Confocal images of leaky gut model counterstained for cell nuclei and cytoskeleton were used to calculate Caco-2 confluency (SI Fig. 1). Values were derived from 10 different co-cultures (≥ 2 fields) of 6 independent experiments.

### 2.7 Dissociation of co-culture into single-cell suspension and analysis for cell size and viability

The release of cells for downstream analysis was performed by enzymatic and mechanical digestion of the co-culture model. First, Caco-2 cell layer was detached by pre-incubation with trypsin-EDTA for 10 min at 37 °C. Cells were collected in Caco-2 medium and placed on ice. Afterwards, co-culture was incubated with collagenase type II solution (12.5 U/mL) for 20 min at 37 °C to release the embedded immune cells from the collagen matrix. Mechanical dissociation was supported by pipetting up and down. The resulting single-cell suspension was run through a 100 μm cell filter and after centrifugation resuspended in PBS. For cell count, an aliquot was analyzed by an automated cell counter (Casy® Model TT; Roche Innovatis AG, Reutlingen, Germany) with a pre-set of distinct size ranges to separate for MUTZ-3 with a size range of 8–13 μm and the other two bigger cell types, Caco-2 and dTHP-1 within 13–50 μm (SI Fig. 2). After sample measurement, size distribution of both size ranges was adjusted to the total volume of cell suspension and represented as total cell yield of 3 wells pooled together.

**Fig. 2:**
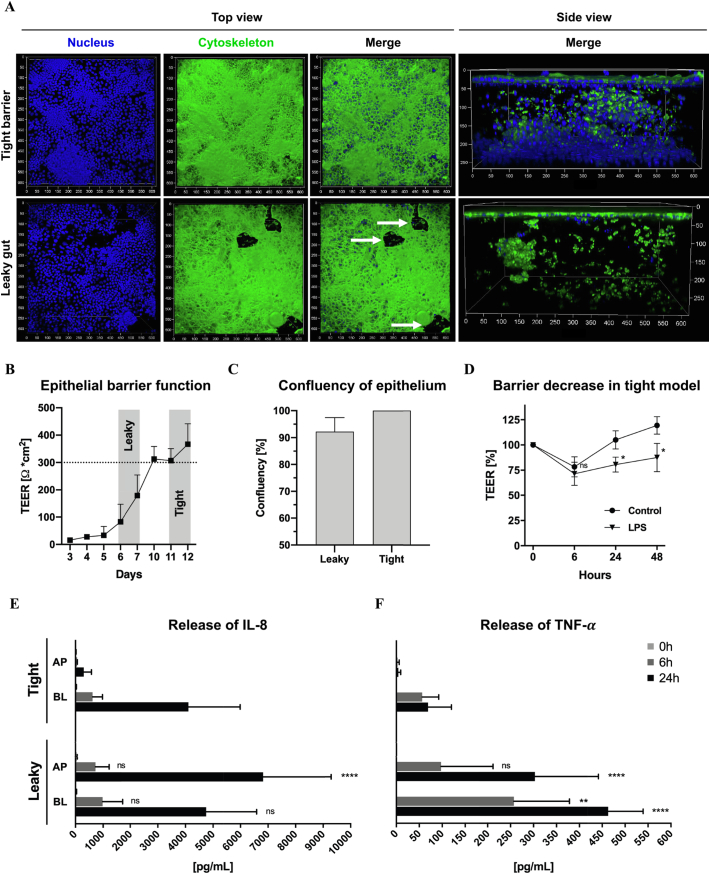
Characterization of leaky gut model with advanced features mimicking IBD condition. Confocal z-stack images of co-cultures representing a tight (day 12; upper panel) or sub-confluent epithelium (day 7; lower panel) stained for cell nuclei (DAPI; blue) and cytoskeleton (AF488-phalloidin; green) (A). Top or side view images are shown in separated or overlaid fluorescence channels (merge) and leaky regions are indicated by white arrows (axis unit = μm). Measurement of epithelial barrier function monitored by TEER over time (intact barrier with 300 Ω*cm^*2*^); (*n* = 6–15; *N* = 4) (B). Confluency measurements of leaky gut model by area calculation of cell-free regions compared to tight epithelium (100% confluency) with ≥2 positions of ten leaky models from 6 independent preparations (*n* = 21; *N* = 6) (C). Barrier function decrease of tight barrier model after LPS inflammation (10 μg/mL) compared to unstimulated control. TEER represented as percentage, normalized to the initial TEER value (*denotes *p* < 0.05 according to Student's test); (*n* = 9; *N* = 3) (D). Comparison of cytokine release after LPS stimulation for tight barrier and leaky gut model monitoring the secretion of interleukin 8 (IL-8) (E) and tumor necrosis factor alpha (TNF-α) (F) to the apical (AP) and basolateral (BL) compartment (** *p* < 0.01 and **** *p* < 0.0001 according to ANOVA); (n = 6; *N* = 2). (For interpretation of the references to colour in this figure legend, the reader is referred to the web version of this article.)

Cell viability was determined by Live/dead cell staining using Calcein AM and DAPI, identifying viable and dead cell fractions, respectively. After incubation with Calcein AM (10 ng/mL in PBS) for 30 min at RT, cells were washed twice with 2% FCS in PBS and resuspended in DAPI solution (0.1 μg/mL in PBS) prior to analysis by FACS. At least 10.000 single cell events were collected and obtained data was analyzed using FlowJo software (FlowJo™ 10.6.0, FlowJo LLC, Ashland, USA). Cells were visualized in granularity *vs.* size (SSC-A/FSC-A) and fluorescence (FITC-A/Pacific blue-A) flow plots or histograms with separated fluorescence channels for living (Calcein AM or FITC-positive) and for dying/dead (DAPI or Pacific blue-positive) cells. Unstained samples or dead cell control (heat-treated; 30 min at 65 °C) were included for the setting of appropriate gating to determine autofluorescence or background and positive control for dead cells, respectively (SI Fig. 3). Respective cell fractions of living (Q1), dead or dying cells (Q2 + Q3), and unstained cell population (Q4) are represented as percentage of total cell count.

**Fig. 3:**
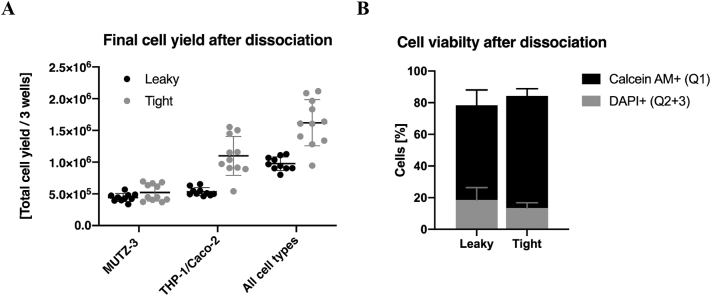
Cell yield and viability of single cell suspension after dissociation of co-culture model. Comparison of cell yield of the resulting single-cell suspensions from leaky gut and tight barrier model using an automated cell counter (3 wells pooled together). Cell size distribution of dendritic-like cells (MUTZ-3; 8-13 μm) and the other two cell types, epithelial (Caco-2) and macrophage-like cells (dTHP-1), within 13-50 μm, were adjusted to the total volume of cell suspension and represented as total cell yield of 3 wells; (*n* = 8–12; *N* = 4) (A). Resulting single cells suspension was stained for living (Calcein AM) and dying/dead cells (DAPI) to identify viable and dead cell fraction using FACS (tight: *n* = 6; *N* = 2 and leaky: *n* = 9; *N* = 3) (B).

### 2.8 JAK/STAT-targeted siRNA and nanocarriers

JAK1-targeting siRNA was obtained as previously described in Clément et al. [24]. siJAK1 duplexes containing 3′-cholesterol modification on the sense strand were synthesized and purified by RNAse-free HPLC at IDT Technologies (Coralville, USA). Lipid nanoparticles (LNPs) with some cationic lipid component and polyethylene glycol (PEG) surfactants [[25], [26], [27]] were used for siRNA delivery and fluorescent label (DiI dye) added [28,29], when indicated. For localization studies, 3´end of the anti-sense strand was ordered additionally with an AF647-fluorescence label and for efficacy testing, a non-targeting siRNA with negative control anti-sense sequence (siNEG). LNP loading with siRNA was performed by simple mixing using N/P ratio 8 which defines siRNA molecules number per nanocarrier. The transfection protocol used herein was optimized within the EU project NEW DEAL consortium.

LNPs were characterized for hydrodynamic diameter and zeta potential using dynamic light scattering (DLS) and electrophoretic light scattering (ELS), respectively (ZetaSizer Nano ZSP; Malvern Instruments, Malvern, UK). For measurements, LNP dispersion was diluted in PBS (DLS) or MilliQ water (ELS) with a final concentration of 70 μg/mL LNPs and 1 mM NaCl (Table 1).

**Table 1:** Characterization of lipid nanoparticles (LNPs).

	LNP concentration [mg/mL]	DiI concentration [mM]	Hydrodynamic diameter [nm]	Poly dispersed index (PDI)	Zeta potential [mV]
Label-free LNPs	238	–	34.7 ± 0.16	0.154 ± 0.014	+27.1 ± 2.3
DiI-labeled LNPs	227	0.188	35.3 ± 0.31	0.176 ± 0.005	+35.4 ± 1.9

### 2.9 Application and evaluation of JAK inhibitors and nanocarriers

Treatment of co-culture model with JAK inhibitors and controls was performed twice for 6 h on two subsequent days, as shown in Fig. 1B/C. siRNA-loaded LNPs (nanoplexes; N/P ratio of 8) at two different concentrations with LNPs at 45 μg (Low) or 90 μg (High) per well with siJAK1 at 135 or 270 nM, respectively. Nanoplexes were applied apically dispersed in antibiotic-free serum-reduced cell culture medium (DMEM 3% FCS 1% NEAA). JAK inhibitor TOFA was applied to both compartments of the co-culture at 5 μM concentration (20 mM DMSO stock). Control treatments were blank LNPs (90 μg/well) or naked siJAK1 (270 nM), LNPs loaded with non-targeting siRNA (siNEG; 270 nM), and commercially available transfection reagent lipofectamine RNAiMAX (Lipo) without or with siJAK1 (270 nM).

#### 2.9.1 Uptake of siJAK1-loaded LNPs

For localization studies, fluorescently-labeled nanocarrier (DiI-LNPs) and cargo (AF647-siJAK1) were used for microscopic analysis, as illustrated in Fig. 1B. Following treatment, cells were fixed and counterstained for cytoskeleton and cell nuclei (2.6). Untreated co-culture (without nanoplexes) served as a control for CLSM settings to avoid unspecific background signal (SI Fig. 4/5A). Fluorescence of LNPs and siJAK1 was detected using HyD detectors with emission filters between 570 - 600 nm (561 nm laser) and 660–685 nm (633 nm laser), respectively. Recorded z-stacks are represented in top or side view with merged fluorescence signals or in split channels for DiI-LNPs (red) and AF647-siJAK1 (cyan).

**Fig. 4:**
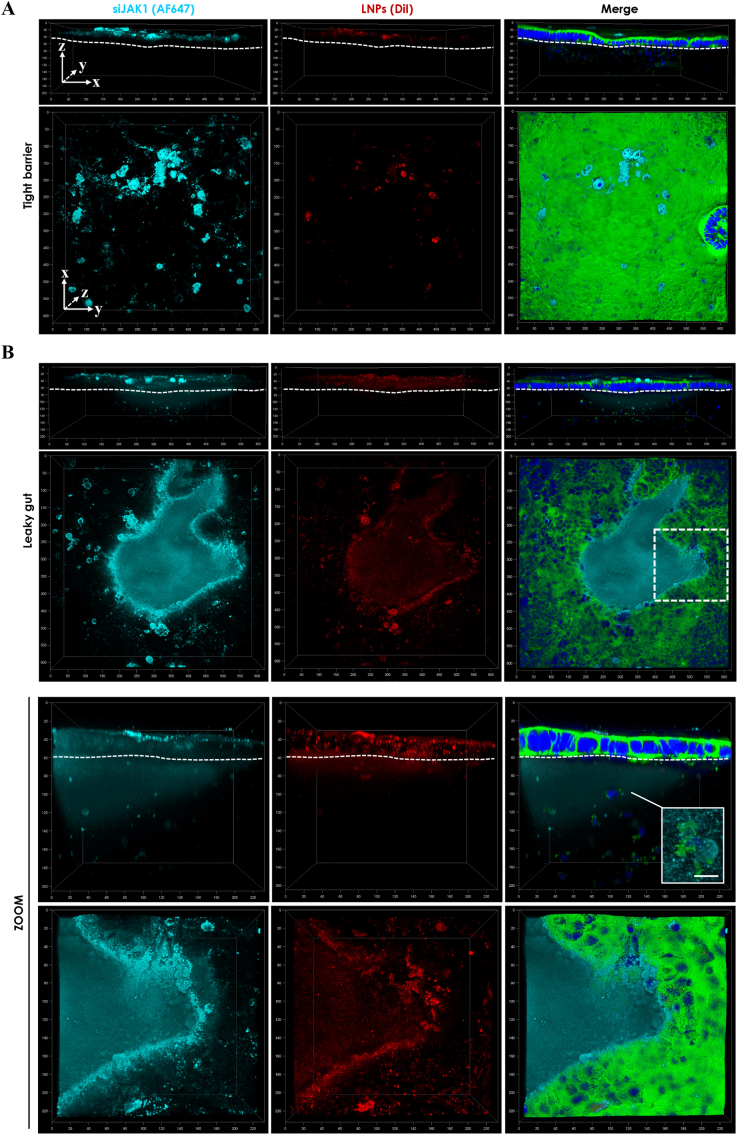
Confocal microscopic images of nanoplex localization in tight barrier (A) and leaky gut model (B). Nanoplexes from fluorescently-labeled LNPs (DiI-label) and JAK1-siRNA (AF647-label) were used. Co-culture was incubated twice apically for 6 h with nanoplexes (N/P ratio of 8) at 90 μg LNP and 270 nM siRNA per well (N/P 8 High). Closeup of chosen area (white dashed line box) shows detailed nanoplex localization within (sub-)epithelial region in magnified view (ZOOM) and detailed view on immune cell (white box; scale bar = 10 μm). Cells are stained for cytoskeleton (AF488-phalloidin; green) and cell nuclei (DAPI; blue). Recorded z-stacks of around 200 μm depth (axis unit = μm) are represented in top (xy-) or side (xz/yz-dimension) view with overlaid fluorescence signal (merge) or split channels for DiI-LNPs (red) and AF647-siJAK1 (cyan). For side views, the basal site of epithelial cell layer (attached to the collagen matrix) is marked by a dashed line to identify the localization of nanocarrier and/or its cargo. (For interpretation of the references to colour in this figure legend, the reader is referred to the web version of this article.)

Quantification of nanoplex uptake was investigated after co-culture dissociation and resulting cell suspension was stained for living and dying/dead cells (see 2.7) prior to FACS analysis. Cells are represented in granularity *vs.* size (SSC-A/FSC-A) and fluorescence flow plots identifying viable cells (FITC-A/Pacific blue-A) and nanoplex association within the viable cell population (PE-A/APC-A). Percentage of cells containing LNPs and/or siRNA are displayed as Q1 (LNP positive; LNP^+^siJAK1^−^), Q2 (double positive; LNP^+^siJAK1^+^), Q3 (siJAK1-positive; LNP^−^siJAK1^+^), and Q4 (double negative; LNP^−^siJAK1^−^) cell fractions (SI Fig. 6).

#### 2.9.2 Efficacy testing of JAK/STAT inhibitors

Efficacy of JAK/STAT inhibition was quantified by the phosphorylation state of STAT1 (pSTAT1) after IFN-γ stimulation using intracellular flow cytometry, as illustrated in Fig. 1C. First, functional assay for STAT1 activation was adapted to different mono-cultures optimizing IFN-γ and TOFA concentration as well as assay performance (SI Fig. 7). After adaptation, mono-cultures of Caco-2 (100.000/well; day 3), dTHP-1 (200.000/well at 50 nM PMA; day 3), and MUTZ-3 (100.000/well) cells or co-cultures were stimulated with recombinant human IFN-γ (50 ng/mL) for 1 h. Subsequently, cells were detached, or co-culture dissociated (2.7) and resulting cell pellet was directly resuspended in 4% (*v*/v) PFA in PBS and fixed for 20 min at 4 °C. After the washing step with PBS, cells were permeabilized with Perm Buffer III for 30 min at 4 °C before intracellular staining with AF647 mouse anti-phospho-STAT1 (pY701) antibody or corresponding isotype control (AF647 mouse IgG 2a, *κ*) according to manufacturer's specification.

For JAK/STAT inhibition of co-culture, label-free LNPs and siJAK1 were applied as described above (see 2.9), followed by IFN-γ addition (50 ng/mL for 1 h) to both compartments of the Transwell® system by partial medium exchange of 100 μL (apical) and 500 μL (basolateral). After pre-treatment and subsequent IFN-γ stimulation, cells were released by enzymatic and mechanic dissociation and fixed immediately. After permeabilization and pSTAT1 staining, cells were washed with PBS and resuspended in 2% FCS in PBS prior to FACS analysis (10.000 cell counts). Cell population was first identified based on its granularity and relative size (SSC-A/FSC-A). Cell aggregates were excluded on the basis of FSC-Height *vs.* -Area (FSC-H/FSC-A) and the single cell gate. Phosphorylation profile of STAT1 (pSTAT1) was represented as histogram with AF647 emission collected at APC instrument setting and summarized in both percentage and the mean fluorescence intensity (MFI) of pSTAT1-positive cells. For comparison of different treatments, pSTAT1-positive cells are represented as percentage, normalized to the IFN-γ stimulated untreated control (100%).

### 2.10 Data analysis and statistics

Data is represented as mean ± standard deviation (SD) and data collected or exported in Microsoft Excel. The graphs are plotted using GraphPad Prism software (Version 9.0.0). If not stated otherwise, all procedures were conducted at least in three independent experiments (N) and measured in technical triplicate (n). Statistical analysis was performed by unpaired Student's *t*-test or one-way analysis of variance (ANOVA) with post-hoc Tukey's test for multiple comparison. Differences were considered as significant for *p* < 0.05 (*), *p* < 0.01 (**), *p* < 0.001 (***), and *p* < 0.0001 (****) and not significant for *p* ≥ 0.05 (ns).

## 3 Results

### 3.1 Characterization of leaky gut model representing IBD conditions

The leaky gut model based on a co-culture of epithelial and immune cells arranged in a physiologically relevant microenvironment aims to mimic human IBD pathophysiology of the intestinal mucosa *in vitro*. Collagen-embedded macrophage- and dendritic-like cells are placed in a Transwell® system and covered with epithelial cells, as shown in Fig. 1A. The leaky gut model reproduces pronounced epithelial damage related to chronic inflammation of IBD associated with an increased loss of barrier integrity beyond tight junction (TJ) openings. Accordingly, inflammation was introduced to the sub-confluent epithelium before reaching confluency. Both, the leaky barrier of the epithelium and inflammatory response mediated by sub-epithelial immune cells provided two essential characteristics of IBD pathophysiology.

First, we investigated cell morphology and spatial features of the co-culture model using confocal microscopy. Fig. 2A shows the differences between tight barrier and leaky gut model. Shortening the cultivation time from previous 12 to 7 days resulted in a sub-confluent layer of epithelial cells with leaky regions (Fig. 2A; white arrows) and reduced cell density of proliferative-active sub-epithelial immune cells (Fig. 2A; side view). We observed cellular adhesion and spreading of both immune cell types and pseudopodial extensions within the collagen matrix (SI Fig. 8). Cell morphology and activation state of macrophage-like cells was analyzed by flow cytometry or confocal microscopy. Upon PMA differentiation, dTHP-1 cells showed increased macrophage (CD11b) and reduced monocyte (CD14) marker expression compared to monocytic THP-1 cells (SI Fig. 9A/B).

Epithelial barrier function of Caco-2 cells growing on the collagen surface was determined over time by transepithelial electrical resistance (TEER) measurements. The leaky gut model showed resistance between 83 ±  64 and 179 ±  76 Ω*cm^2^ for day 6 and 7 after seeding, while the densely grown cell layer of the tight barrier model generated values above 300 Ω*cm^2^ (Fig. 2B). Furthermore, surface area measurements of sub-confluent regions within the leaky gut model resulted in a confluency of 87–97% compared to the tight epithelium (Fig. 2C).

Further characterization involved the responsiveness to *E. coli* lipopolysaccharides (LPS). For the tight barrier model with an established epithelial barrier, LPS stimulation leads to a significant decrease of TEER to about 80 ±  7% after 24 h compared to unstimulated control (Fig. 2D). Both co-cultures showed an increased cytokine production including interleukin-8 (IL-8) and tumor necrosis factor-alpha (TNF-α), but differences in immune response merely after 24 h of stimulation. Similar amounts of IL-8 were released after 6 h to the basolateral site (BL), with 980 ±  750 pg/mL and 620 ±  350 pg/mL for leaky gut and tight barrier model, respectively (Fig. 2E). The same was observed for the apical compartment (AP), with 720 ±  500 pg/mL and 60 ±  10 pg/mL. After 24 h, besides a comparable release of IL-8 for BL, a significantly higher secretion was found for AP with 6800 ±  2500 pg/mL for the leaky gut compared to only 300 ±  140 pg/mL for the tight barrier model. Finally, we detected a general higher production of TNF-α within the leaky gut model for both time points and compartments. A significantly higher response was observed after 24 h with 300 ±  140 pg/mL (AP) and 460 ±  80 pg/mL (BL) compared to very low amounts for the tight barrier model with the highest release of 70 ±  50 pg/mL (BL) (Fig. 2F).

### 3.2 Characterization of cells after dissociation of co-culture model

The dissociation of the co-culture was necessary to perform downstream analysis including the interaction with the drug/delivery system. After pre-determined cultivation and treatment, co-culture model was dissociated using a combination of enzymatic digestion and mechanic disruption. Resulting single-cell suspension was characterized in terms of total cell yield using an automated cell counter with pre-set size ranges to discriminate between cell debris and living cells as well as their cell type. As anticipated, a lower number of cells was collected from the leaky gut model (day 7) compared to the tight barrier model (day 12) (Fig. 3A). Single-cell suspension from the leaky gut model contained approximately 1 × 10^6^ cells (from three wells pooled together) consisting of an equal amount of MUTZ-3 and THP-1/Caco-2 cells. In comparison, cells from the tight barrier model showed a higher cell yield after dissociation with around 1.7 × 10^6^ cells with a similar proportion of MUTZ-3 cells, but more than a double amount of the other cell types. As cell counting of heterogenous cell population is limited to display the cell size distribution for each specific cell type, only an estimation of cell proportion was done. Cell count measurements were performed first for each individual cell line separately in order to identify the size-related differences showing mean diameters of around 10 μm for MUTZ-3, 16 μm for monocytic THP-1, 19 μm for Caco-2, and 24 μm for macrophage-like (dTHP-1) (SI Fig. 2A). Accordingly, dendritic cells (MUTZ-3) with a small and narrow size distribution of 8–13 μm were distinguishable from the other cell types, comprising (d)THP-1 and Caco-2 cells, with similar size range between 13 and 30 μm (SI Fig. 2B).

Besides a sufficient cell number, the cell survival after co-culture disaggregation has an important impact on subsequent cell analysis. Therefore, cell viability of single-cell suspensions was investigated using Calcein AM and DAPI staining to identify living (FITC-positive; Q1) and dead or dying cell fractions (Pacific blue-positive; Q2 + 3), respectively (Fig. 3B). In summary, the majority of cells showed good viability for both co-culture models, with 78.4 ±  9.6% for the leaky gut and 84.3 ±  4.5% for the tight barrier model (Fig. 3B).

### 3.3 Trafficking and localization of siRNA-loaded nanomedicine

In order to investigate nanoplex delivery into the human intestinal mucosa after oral administration, fluorescently-labeled nanocarrier (DiI-LNPs) and cargo (AF647-siJAK1) were applied to the apical site of the co-culture model. The effect of epithelial confluency and disease-related barrier dysfunction was evaluated on nanoplex absorption and penetration to the underlying immune cells. Accordingly, leaky gut model was treated with nanoplexes for six hours for two consecutive days, starting from day 6 and in comparison, tight barrier model with a confluent Caco-2 cell layer was treated from day 11.

Fig. 4 shows confocal microscopic images of nanoplex localization in tight barrier and leaky gut model at high concentration of 90 μg LNPs and 270 nM siJAK1 per well. For comparative purposes, side or orthogonal views of the co-culture revealed the penetration of nanoplexes into the intestinal tissue while top views provide an overview of nanoplex absorption to the epithelial surface. Nanoplex-treated tight barrier model showed a co-localization within the epithelial cell layer where nanocarrier and cargo were taken up by Caco-2 cells but did not cross the epithelial cell layer to reach the underlying immune cells (Fig. 4A). The same was observed for low nanoplex concentration (45 μg LNPs and 135 nM siJAK1 per well; N/P 8 Low) and control treatments with blank LNPs (SI Fig. 4B/C). In contrast to the tight barrier model, nanoplexes were accumulated with the leaky regions and started to penetrate into the sub-epithelial layer (Fig. 4B). The closeup view of the sub-confluent region reveals that both, LNPs and siRNA, were localized within the Caco-2 cells and showed increased diffusion through the leaky areas and direct contact with underlying immune cells (Fig. 4B; ZOOM). Increased nanoplex penetration into the co-culture was observed for the higher concentration of nanoplexes with around 200 μm depth (Fig. 4B) in comparison to the lower concentration with approximately 100 μm (SI Fig. 5B). The application of blank LNPs which have a more cationic charge in comparison to siRNA-loaded LNPs, showed a comparable penetration into the leaky regions and facilitated cellular interaction (SI Fig. 5C). As expected, naked siRNA at high concentration (270 nM) was detected for both co-culture models only in traces on the surface of the epithelial cell layer and due to its anionic charge showed low cellular interaction (SI Fig. 4/5D). In order to exclude the possible effect of the collagen matrix to impede nanoplex diffusion into the sub-epithelial space, the penetration was performed in a co-culture without an epithelial cell layer on top. Both, nanocarrier and siRNA, were able to diffuse through the collagen matrix with advanced penetration of more than 300 μm depth after 6 h of incubation and showed uptake within the underlying immune cells (SI Fig. 10A/B). In comparison, the application of polystyrene nanoparticles of 100 nm size displayed comparable results, despite the majority remained attached to the surface of collagen matrix (SI Fig. 10C).

**Fig. 5:**
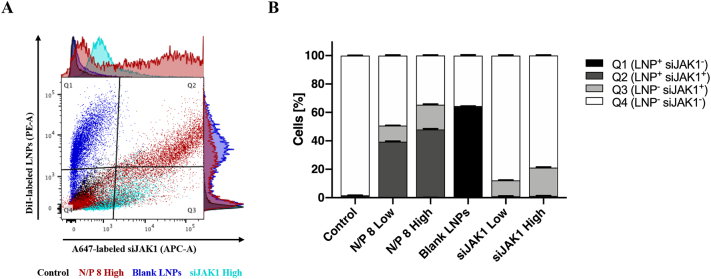
Flow cytometric quantification of cellular association of nanoplexes in leaky gut model. Leaky gut model was treated with fluorescently-labeled nanocarrier (DiI-LNPs) and siRNA (AF647-siJAK1). Co-culture was incubated twice apically for 6 h with nanoplexes (N/P ratio of 8) at two different concentrations with LNPs at 45 μg or 90 μg per well and siJAK1 at 135 nM (Low) or 270 nM (High), respectively. Control treatments were co-cultures without nanocarrier and siRNA (Control), blank LNPs (90 μg/mL) or naked siJAK1 (Low/High). After co-culture dissociation, the resulting cell suspension was stained for living (Calcein AM; 10 ng/mL) and dying/dead cells (DAPI; 0.1 μg/mL). Density plot overlay shows nanoplex association within the viable cell population (PE-A/APC-A; right column) (A). Percentage of cells containing LNPs and/or siRNA are displayed as Q1 (LNP positive; LNP^+^siJAK1^−^), Q2 (double positive; LNP^+^siJAK1^+^), Q3 (siJAK1-positive; LNP^−^siJAK1^+^), and Q4 (double negative; LNP^−^siJAK1^−^) cell fractions, summarized in a stacked graph (B). Data is shown as mean ± SD of triplicates of one experiment (*n* = 3; *N* = 1).

Fig. 5 shows preliminary quantification of cellular association of nanoplexes that was analyzed by flow cytometry after co-culture dissociation. Cellular adhesion and uptake of nanocarrier and cargo are represented by the percentage of positive cells within the viable cell population (Fig. 5A). For nanoplexes, a concentration dependent increase of cellular association was observed with 39% of double- and 11% of siJAK1-positive cells for the low and 48% of double- and 17% of siJAK1-positive cells for the high dose (Fig. 5B). The application of blank nanocarriers at high concentration showed 64% of LNP-positive cells, whereas naked siJAK1 displayed 11% and 20% of siJAK1-positive cells for low and high dose, respectively.

### 3.4 Efficacy testing of siRNA-loaded nanomedicine targeting JAK/STAT signal pathway

The inhibition of JAK/STAT signal pathway was performed by two different pharmaceutical approaches using JAK inhibitors (JAKi): i) TOFA, a small molecule pan-JAKi with broad mechanism of action, but preferentially inhibiting JAK1/3, and ii) siJAK1 as a siRNA-based compound with high specificity for JAK1. The downregulation of JAK/STAT signaling was evaluated by the pSTAT1 after IFN-γ stimulation. Downstream transcriptional effects of JAK1 inhibition were quantified using phospho-specific antibody staining.

**Fig. 6:**
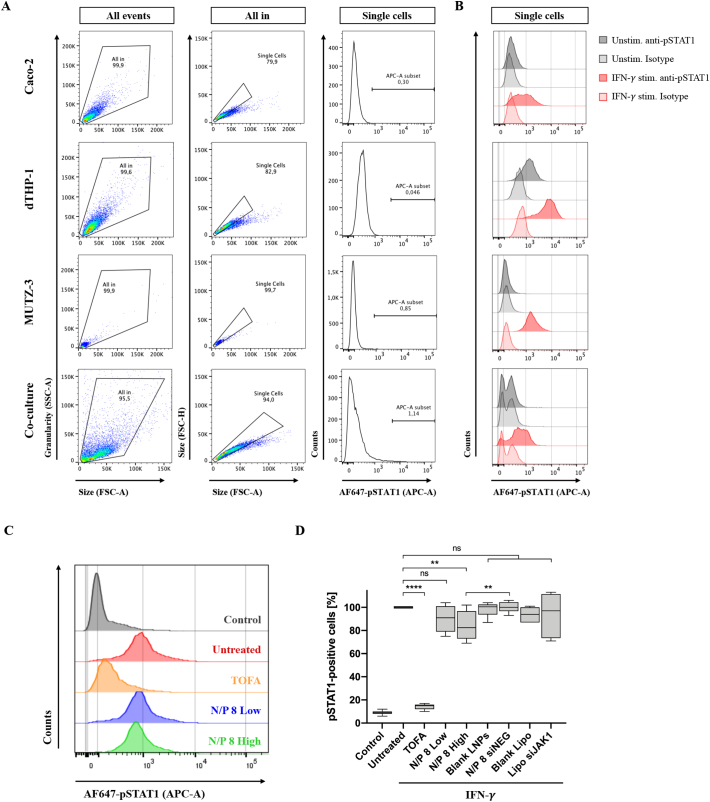
Flow cytometric analysis of JAK/STAT signal inhibition in leaky gut model. Mono-culture of Caco-2, dTHP-1, and MUTZ-3 cells or co-culture model were either left unstimulated or stimulated with IFN-*γ* (50 ng/mL) for 1 h. Cells were detached (mono-cultures) or dissociated (co-culture), fixed and permeabilized for intracellular staining with AF647 mouse anti-phospho-STAT1 (pY701) antibody or corresponding isotype control (AF647 mouse IgG 2a, *κ*). Flow plots show the gating strategy of unstimulated unstained controls for each cell line and co-culture model (A). Phosphorylation profile of STAT1 (pSTAT1) represented as histogram with percentage of pSTAT1-positive cells. The overlay of histograms summarizes (un-) stimulated controls stained either with specific pSTAT1 antibody (solid line) or isotype control (dashed line) (B). For JAK/STAT inhibition, the leaky co-culture model was pre-treated with TOFA (5 μM) or siJAK1-loaded nanoplexes (N/P ratio of 8) at low and high concentration (45 μg or 90 μg LNPs and 135 or 270 nM siRNA per well, respectively). Following the second 6 h sample incubation time, co-culture was IFN-*γ* stimulated, dissociated, and immediately stained and analyzed. Histogram overlay shows unstimulated untreated control (grey) and IFN-*γ*-induced pSTAT1 without anti-inflammatory treatment (untreated; red) or after treatment with TOFA (orange), or nanoplexes at low (blue) and high (green) concentration (C). Further control treatments included blank LNPs (90 μg/well); LNPs loaded with non-targeting siRNA (siNEG; 270 nM) and lipofectamine RNAiMAX (Lipo) alone or with siJAK1 at 270 nM concentration. pSTAT1-positive cells are represented as percentage, normalized to the untreated cells (D). The box plots depict the minimum and maximum values (whiskers), the upper and lower quartiles, and the median. Significant differences are represented as ** *p* < 0.01 and **** *p* < 0.0001 according to ANOVA; for TOFA (*n* = 15; *N* = 5), N/P 8 Low/High (*n* = 9; *N* = 3), and control treatments (*n* = 6; *N* = 2). (For interpretation of the references to colour in this figure legend, the reader is referred to the web version of this article.)

First, we investigated for each cell type of the co-culture model separately the responsiveness to IFN-γ and related STAT1 activation that is characterized by its phosphorylation state. Fig. 6 summarizes the different pSTAT1 activation profiles of all three cell types including Caco-2, dTHP-1, and MUTZ-3 cells, resulting in different degrees of pSTAT1-positive cells within the corresponding cell population in comparison to the co-culture. Accordingly, quantification of STAT1 phosphorylation of mono- or co-cultures was detected by the APC fluorescence shift within the single cell selection and showed no background derived from auto-fluorescence for unstimulated cells without the addition of antibodies (Fig. 6A). Assay performance was validated by the use of appropriate controls including (un-)stained or (un-)stimulated cells and isotype controls and showed high specificity of respective phospho-specific antibody staining without fluorescence background signal (Fig. 6B). Different concentrations of IFN-γ were tested in 2D mono-cultures and the highest concentration (50 ng/mL) was chosen to ensure the activation of JAK/STAT pathway for both, mono- and co-culture setups (SI Fig. 7). Upon IFN-γ stimulation, epithelial cells showed a reduced activation of JAK/STAT signal pathway with around 30% of pSTAT1-positive cells compared to unstimulated control (Fig. 6B; upper panel). Whereas both immune cell types were highly responsive to IFN-γ displaying approximately 60% (dTHP-1) and almost 90% (MUTZ-3) of STAT1 phosphorylation (Fig. 6B). The overall level of pSTAT1 from IFN-γ-stimulated leaky gut model, a mixture of all three cell types collected after dissociation, revealed approximately one third of pSTAT1-positive cells (32 ± 1%) compared to unstimulated control (SI Fig. 7C). In comparison, less pSTAT1-positive cells (11 ± 2%) were detected from IFN-γ-stimulated tight barrier model (SI Fig. 7D).

The efficacy for JAK/STAT downregulation within the leaky gut model was investigated by different JAKi and mechanisms of actions. Fig. 6C/D illustrated the flow cytometry results obtained after different pre-treatment strategies applied to the leaky gut model including TOFA and two nanoplex concentrations. Therapeutic efficacy was compared to IFN-γ stimulated untreated control (untreated; red), whereas unstimulated untreated control showed the basal phosphorylation level of STAT1 (control; grey). The small molecule JAK inhibitor TOFA was added from both sites to the apical and basolateral compartment at 5 μM. JAK/STAT signaling was inhibited by 86 ± 2% after TOFA pre-treatment, as indicated by the quantified fluorescence signal almost back to baseline (TOFA; orange). Treatment with LNP carrier and JAK1 targeting siRNA showed dose-dependent JAK/STAT inhibition of 10 ± 12% and 16 ± 12% of signal inhibition for low and high nanoplex concentrations respectively, while Lipofectamine was not effective (Fig. 6D). Low nanoplex concentration (N/P 8 Low; blue) attenuated the IFN-γ-induced phosphorylation of STAT1, but without significant difference to untreated cells. However, the doubling of nanoplex concentration (N/P 8 High; green) showed significant inhibition of STAT1 activation compared to IFN-γ stimulation alone (untreated). Both nanoplex concentrations displayed high standard deviations with around 12% without significant changes in STAT1 phosphorylation status between the treatments. The observed JAK/STAT down-regulation was associated with the silencing efficacy of siJAK1, since control treatments with blank LNPs (without siRNA) or loaded with non-targeting siRNA (siNEG) at comparable concentrations did not affect signal transduction with 98 ± 6% and 100 ± 5%, respectively (Fig. 6D).

## 4 Discussion

In the context of IBD and pharmaceutical development of novel anti-inflammatory compounds, *in vitro* intestinal models aim to provide representative and predictive platforms for optimizing drug delivery and therapeutic efficacy. These models are ranging from simplified 2D cell cultures of the intestinal barrier and more challenged 3D co-cultures using primary cells or microfluidic devices with dynamic flow and peristaltic motion to improve physiological resemblance [14]. Nevertheless, increased complexity retains some limitations including limited access and ethical aspects regarding primary tissue, the lack of immune-related responses, and high variability between individual samples [16,17]. Moreover, there is an unmet need for appropriate *in vitro* models to evaluate drug interaction within the human intestinal mucosa under diseased conditions, especially for IBD-related pathophysiology. In particular, organoid-based intestinal models generated from human patient biopsies are reflecting the genetic signature of the original epithelium as well as the epithelial cell complexity to a higher extent [17]. The here presented leaky gut model is based on human cell lines to maximise the model's applicability to a human exposure scenario and to allow for better standardization and validation, as well as cell accessibility throughout other laboratories. Correspondingly, as a screening model for nanoparticles targeting sub-epithelial immune cells and together with the desired introduction of well-controlled epithelial breaches, the implementation of organoid cell technology seems to be exaggerated in this context.

The leaky gut model mimics both, a dysfunctional epithelial barrier and inflammatory processes necessary for the evaluation of distinct therapeutic outcomes. IBD pathogenesis is associated with an increased intestinal permeability and local inflamed microenvironment with elevated concentration of cytokines and immune cell infiltration [30]. The leaky gut is often referred to altered permeability with enhanced paracellular diffusion of luminal contents and moreover, related to epithelial gaps or breaches resulting from an impaired epithelial cell turnover to replenish cells after cellular damage or apoptosis [[31], [32], [33], [34]]. In contrast to that, the leaky gut model is supported by the observation of endoscopic analysis in IBD patients showing structural defects (microerosions) of the injured gut barrier and correlated mucosal wound healing [[35], [36], [37]]. Modelling these excessive epithelial damages was realized by the leaky gut model and the introduction of a sub-confluent Caco-2 layer. This guaranteed the presence of small openings within the enterocyte-like layer mimicking a compromised epithelium necessary to perform drug absorption studies under diseased conditions. Electrophysiological quantification of epithelial barrier tightness is indicative for the presence of differentiated cells and the TJ expression regulating paracellular permeability and integrity [38]. Accordingly, TEER measurements of the leaky gut model displayed resistance values below 200 Ω*cm^2^, representing a not fully established but partial epithelial barrier. This observations are in line with previous classification, where GI epithelial barrier is considered ´tight´ with resistance around 2000 Ω*cm^2^, ´intermediate´ with values between 300 and 400 Ω*cm^2^, and ´leaky´ ranging from 50 to 100 Ω*cm^2^ [39]. However, depending on the Caco-2 cell clone and related culture conditions such as seeding density or serum content, cell growth-related variations may occur that affect the development of the epithelial barrier. Furthermore, Caco-2 cell growth is influenced by the passage number showing increased proliferation rate as well as TEER towards higher passages [40]. In line with previous reports, we observed that Caco-2 cells grew slower on top of the collagen gel compared to cell growth on Transwell® filters [41,42]. Considering these variabilities, we used consistently the same passage numbers and avoided late passages to receive a comparable degree of Caco-2 sub-confluency generating an approximately 72 h time window for co-culture treatment. Due the fact that the leaky gut model is based on the co-culture of human cell lines, it's main limitation includes the immortalized or cancerous nature of these cells which do not represent the expression profile of normal tissue. In this regard, Caco-2 cells are limited for the investigation of transporter-mediated membrane transport and metabolism, as well as represent different epithelial tightness [43]. In contrast to organoid cell cultures, the leaky gut model is not suitable to elucidate the transport across the epithelial barrier and its underlying mechanisms.

Compared to previous co-cultures models that used inflammatory agents to mimic inflammation [20,44], we optimized the leaky gut model in terms of pro-inflammatory response related to activated immune cells and cytokine production. Interestingly, a significantly higher cytokine production (IL-8 & TNF-α) was observed for the leaky gut model after LPS stimulation, despite a reduced total cell number. Compared to the tight model, the increased cytokine detection in the apical compartment of the leaky gut model could be related to the higher permeation of LPS into the sub-epithelial layer leading to an increased immune cell activation. In earlier studies, macrophage-like cells (dTHP-1) were identified as the main contributors for cytokine production, however, TNF-α expression was detected only at the transcriptional level [19]. Besides the possible effect of cytokine entrapment or retention within the collagen matrix, the detection of ongoing inflammation was comparable with other co-culture models [45]. Compared to previous protocol, we increased PMA concentration up to 4-fold and embedded the double amount of macrophages in the leaky gut model [20]. According to accepted criteria to recognize fully differentiated THP-1 cells, dTHP-1 cells showed elevated CD11b expression necessary for cell adhesion and motility, and reduced monocyte marker CD14 expression usually downregulated during PMA-differentiation [23,46]. Enhanced secretion of pro-inflammatory cytokines within the leaky gut model could be explained with the a more activated macrophage phenotype due to higher and longer exposure to PMA, which concurs with previous reports [47]. Moreover, doubling the number of dTHP-1 cells and shorter cultivation time of the leaky gut model turned out to be advantageous for providing more active and differentiated dTHP-1 cells and for studying LPS-stimulated immune response compared to the tight barrier model. Previous studies have shown that an extended culture of dTHP-1 cells in the absence of PMA will lead to de-differentiation and recovery of proliferative activity generating monocytic THP-1 cells [46].

In contrast to preceding applications of the co-culture model including indirect interrogation between healthy and diseased conditions [20], the dissociation of the co-culture was performed in order to provide novel insights into cell number and viability as well as the feasibility of downstream analysis. Cell counter analysis gave an estimation of the total cell yield with a sufficient number of viable cells collected for downstream analysis. As mentioned above, the potential presence of de-differentiated THP-1 cells may contribute to the overall cell ratio. Regarding the doubling time of each cell type present, MUTZ-3 cells are the slowest proliferating cells (100−120h), THP-1 the fastest (~35–50 h) and Caco-2 cells in the middle range (~60 h) (data sheets from DMSZ/ATCC). Taking into account that collagen embedding could alter the cell behaviour and function of cells, information about the doubling time for each cell line are indicative and may not represent cell growth under co-culture conditions [48]. Thus, cell size distribution may not be a specific determinant for cell number evaluation of this heterogenous cell suspension as dead or dying cells may fall in the sized range of smaller cell types and cell aggregates may be identified as cells with a bigger shape. Based on clinical observations, where intestinal inflammation is altering the numerical and proportional composition of intestinal immune cells, comprising tissue-resident and recruited monocyte-derived macrophages of dendritic cells, the leaky gut model aims to reproduce these changes [49,50].

The investigation of trafficking and cellular interaction of nanoplexes revealed the accumulation of both, siRNA and LNPs, within the sub-confluent regions. LNPs aim to deliver the siRNA therapeutics locally into the inflamed gut without affecting the surrounding healthy tissue in IBD patients, which is related to the concept of passive targeting to inflamed tissue, comparable to the concept of epithelial enhanced permeation and retention effect [51]. In previous uptake studies, LPS-stimulation of the tight barrier model and resulting barrier decrease of 20% did not improve LNP penetration into the sub-epithelial space and even for extended incubation of 24 h, nanocarriers were not able to reach the underlying immune cells (SI Fig. 11). Contrary to this, the advantage of the leaky gut model is the reproduction of the observed phenomenon *in vivo* with nanoparticle accumulation within inflamed intestinal breaches. In this regard, the leaky gut model provided the study of paracellular transport across these epithelial openings, hence, intercellular junctions as well as TJ expression level were not decisive at that point. Nevertheless, a significant contribution to drug carrier accumulation within the inflamed tissue is mediated through the cellular uptake by phagocytes, such as resident or recruited macrophages, neutrophils or dendritic cells that are enriched within the inflamed mucosa [52]. LNPs designed for intracellular delivery were successfully internalized by Caco-2 cells together with siRNA and in parts accumulate faster in dead or dying cells due to cell membrane leakiness. As cellular uptake is affected by the physiochemical property of the carrier, present LNP formulation was beneficial for cellular uptake due to cationic charge and 40 nm size. Despite these properties may change once LNPs are subjected to biological fluids, PEG coating reduces LNP hydrophobicity and surface charge density [53]. Under physiological conditions, nanoplexes should overcome the mucus barrier before reaching the intestinal epithelium. The high density of PEGylation on LNP surface favours mucus penetration, a rather important biological barrier for nanocarriers [54]. First attempts showed an increased percentage of siJAK1-positive cells when complexed with LNPs, however, delivery efficacy and target engagement against siJAK1 was found to be a more important readout compared to cellular association of nanoplexes.

Localized delivery of JAK inhibitors and corresponding drug function was investigated within the leaky gut model that combines an intestinal barrier and immune target cells. Transcriptional data from human biopsis showed an upregulation of all four JAK kinases in IBD patients suffering from UC, and JAK1 was found in myeloid and epithelial cells of the human intestine (predominantly macrophages and dendritic cells) [55]. However, JAK/STAT inhibition should be highly specific to not interfere with other biologically relevant processes such as cell differentiation, growth and survival [56]. This requires a local and selective JAK inhibition, limited to the inflamed tissue with minimized systemic exposure and preferably restricted to one specific JAK for selective immune modulation within target cell populations [55,57]. For that, model suitability was tested in terms of adequate response to IFN-γ for JAK/STAT activation and quantified *via* STAT1 phosphorylation (pSTAT1) downstream of the signaling cascade. Compared to Western blotting, flow cytometric detection and quantification of pSTAT1 increases the resolution down to individual cells [58]. Assay performance was validated using isotype and more important unstimulated controls to define antibody specificity and to identity basal phosphorylation level for each cell type, respectively. Macrophages showed the highest amount basal phosphorylation compared to the other cell types, which has been attributed to the PMA-mediated expression of an IFN-γ-inducible enzyme that may have lead the expression in dTHP-1 cells [59]. In line with previous studies, transient IFN-γ-mediated STAT1 was activated in macrophages typically within 15–60 min with an resolution after 2–6 h after stimulation [60]. However, STAT phosphorylation is highly dynamic and fast de-phosphorylation requires fast cell fixation [58]. Accordingly, co-culture dissociation right after IFN-γ-stimulation may have an impact on the detection of the phosphorylation state due to postponed fixation step. Significant JAK/STAT inhibition using TOFA is related to the fact that the drug may have reached the majority of cells due to increased permeation into the co-culture model. TOFA is the first pan-JAKi, preferentially inhibiting JAK1/3, approved for UC and diffuses passively inside the cells and impedes downstream phosphorylation of target STATs [61,62]. TOFA was used here as a positive control and repetitive dosage applied to both compartments ensured the saturation of the system. A recent study used the same concentration and revealed a high *in vitro* permeability in Caco-2 cells with a low efflux ratio [63]. LNPs, composed of a lipid core and stabilized by a surfactant shell, were used to improve siRNA delivery. Negatively charged siRNA targeting human JAK1 was complexed on the surface of cationic LNPs. However, in comparison to TOFA, the silencing effects of siJAK1 were less pronounced but correspond to the observed siRNA trafficking within the leaky gut model where nanoplexes accumulated within the leaky regions. After their penetration into the sub-epithelial layer only a small portion of the underlying immune cells was reached by siRNA, as the main protagonist of pSTAT1 activation. The sub-confluent and still proliferative active Caco-2 cell layer displayed the internalization of siRNA-loaded LNPs to a higher extent, but Caco-2 cells were less responsive to IFN-γ and therefore could contribute less to the overall phosphorylation pattern of STAT1. Moreover, transfection of differentiated epithelial cells is regarded as more difficult compared to less differentiated cells [64]. In conclusion, this data suggests that a much higher percentage of cells within the co-culture must internalize siRNA-loaded LNPs to induce higher JAK/STAT inhibition. Nevertheless, to improve the resolution and understand which cell type contributes to what extent to the overall phosphorylation profile, phospho-specific staining could be combined with cell-type specific marker, such as epithelial and immune cell marker. Furthermore, as wound healing and inflammation are connected biological processes [30], the still growing epithelial cell layer of the leaky gut model could be further investigated regarding novel targets and intestinal tissue repair.

## 5 Conclusion

In summary, we have established a complex *in vitro* model of the chronically inflamed human intestinal interface for studying anti-inflammatory drugs and nanomedicines. The 3D cell model developed here comprises a sub-confluent epithelial monolayer with adjacent components of the immune system mimicking severe IBD conditions. Within this system, we provide a functional assay to detect a particular target molecule for the prediction of reliable drug-target interaction. Disease modelling allows to investigate intestinal barrier crossing of siRNA-based nanomedicine under pathophysiological conditions. Accumulation of nanoplexes was observed within the epithelial breaches followed by the penetration to the sub-epithelial layer reaching target immune cell types located underneath. Such disease model should help to estimate expectable efficacy of siRNA nanomedicine and allow a first selection and optimization of delivery systems. Finally, this intestinal *in vitro* model could serve as a pre-clinical platform to complement *in vivo* analysis for faster translation of novel drugs and/or drug delivery systems into the clinic.

## Author contributions

OH, BL, and CML were responsible for the conceptualization, writing and editing. OH performed experiments, analyzed data and wrote the manuscript. AN, DJ, and FN provided lipid nanocarriers and optimized complexation protocol. ES and XG designed and validated siRNA against human JAK1. All authors read and approved the final research article.

## Appendix A Supplementary data

Supplementary material Image 1

## References

[1] Ananthakrishnan A.N. (2015). Epidemiology and risk factors for IBD. Nat. Rev. Gastroenterol. Hepatol..

[2] Zhang Y.Z., Li Y.Y. (2014). Inflammatory bowel disease: pathogenesis. World J. Gastroenterol..

[3] Kaplan G.G., Windsor J.W. (2021). The four epidemiological stages in the global evolution of inflammatory bowel disease. Nat. Rev. Gastroenterol. Hepatol..

[4] Neurath M.F. (2017). Current and emerging therapeutic targets for IBD. Nat. Rev. Gastroenterol. Hepatol..

[5] Park S.C., Jeen Y.T. (2018). Anti-integrin therapy for inflammatory bowel disease. World J. Gastroenterol..

[6] Chan H.C.-H., Ng S.C. (2017). Emerging biologics in inflammatory bowel disease. J. Gastroenterol..

[8] Danese S., Argollo M., Le Berre C., Peyrin-Biroulet L. (2019). JAK selectivity for inflammatory bowel disease treatment: does it clinically matter?. Gut.

[9] Valatas V., Bamias G., Kolios G. (2015). Experimental colitis models: insights into the pathogenesis of inflammatory bowel disease and translational issues. Eur Aust. J. Pharm..

[10] Mizoguchi E., Low D., Ezaki Y., Okada T. (2020). Recent updates on the basic mechanisms and pathogenesis of inflammatory bowel diseases in experimental animal models. Intest Res..

[11] Koboziev I., Karlsson F., Zhang S., Grisham M.B. (2011). Pharmacological intervention studies using mouse models of the inflammatory bowel diseases: translating preclinical data into new drug therapies. Inflamm. Bowel Dis..

[12] Gibbons D.L., Spencer J. (2011). Mouse and human intestinal immunity: Same ballpark, different players; Different rules, same score. Mucosal Immunol..

[13] Wagar L.E., Difazio R.M., Davis M.M. (2018). Advanced model systems and tools for basic and translational human immunology. Genome Med..

[14] Dutton J.S., Hinman S.S., Kim R., Wang Y., Allbritton N.L. (2019). Primary cell-derived intestinal models: recapitulating physiology. Trends Biotechnol..

[15] Batista Leite S., Cipriano M., Carpi D., Coecke S., Holloway M., Corvi R., Worth A., Barroso J., Whelan M. (2021). Establishing the scientific validity of complex in vitro models: Results of a EURL ECVAM. Survey Publ. Off. Eur. Union.

[16] Steinway S.N., Saleh J., Koo B.K., Delacour D., Kim D.H. (2020). Human microphysiological models of intestinal tissue and gut microbiome. Front Bioeng. Biotechnol..

[17] Fatehullah A., Tan S.H., Barker N. (2016). Organoids as an in vitro model of human development and disease. Nat. Cell Biol..

[18] Costa J., Ahluwalia A. (2019). Advances and current challenges in intestinal in vitro model engineering: a digest. Front Bioeng. Biotechnol..

[19] Leonard F., Collnot E.M., Lehr C.M. (2010). A three-dimensional coculture of enterocytes, monocytes and dendritic cells to model inflamed intestinal mucosa in vitro. Mol. Pharm..

[20] Susewind J., De Souza Carvalho-Wodarz C., Repnik U., Collnot E.M., Schneider-Daum N., Griffiths G.W., Lehr C.M. (2016). A 3D co-culture of three human cell lines to model the inflamed intestinal mucosa for safety testing of nanomaterials. Nanotoxicology.

[21] Ahmad R., Sorrell M.F., Batra S.K., Dhawan P., Singh A.B. (2017). Gut permeability and mucosal inflammation: Bad, good or context dependent. Mucosal Immunol..

[22] Parker A., Vaux L., Patterson A.M., Modasia A., Muraro D., Fletcher A.G., Byrne H.M., Maini P.K., Watson A.J.M., Pin C. (2019). Elevated apoptosis impairs epithelial cell turnover and shortens villi in TNF-driven intestinal inflammation. Cell Death Dis..

[23] Lund M.E., To J., O’Brien B.A., Donnelly S. (2016). The choice of phorbol 12-myristate 13-acetate differentiation protocol influences the response of THP-1 macrophages to a pro-inflammatory stimulus. J. Immunol. Methods.

[24] Clément F., Nougarède A., Combe S., Kermarrec F., Dey A.K., Obeid P., Navarro F.P., Marche P.N., Sulpice E., Gidrol X. (2022). Therapeutic siRNAs targeting JAK/STAT signalling pathway in inflammatory bowel Diseases. J. Crohns. Colitis.

[25] Delmas T., Fraichard A., Bayle P.-A., Texier I., Bardet M., Baudry J., Bibette J., Couffin A.-C. (2012). Encapsulation and release behavior from lipid nanoparticles: model study with Nile red fluorophore. J. Colloid Sci. Biotechnol..

[26] Texier I., Goutayer M., Da Silva A., Guyon L., Djaker N., Josserand V., Neumann E., Bibette J., Vinet F. (2009). Cyanine-loaded lipid nanoparticles for improved in vivo fluorescence imaging. J. Biomed. Opt..

[27] Hibbitts A., Lucía A., Serrano-Sevilla I., De Matteis L., McArthur M., de la Fuente J.M., Aínsa J.A., Navarro F. (2019). Co-delivery of free vancomycin and transcription factor decoy-nanostructured lipid carriers can enhance inhibition of methicillin resistant Staphylococcus aureus (MRSA). PLoS One.

[28] Gravier J.J., Garcia F.P.N.Y., Delmas T., Mittler F., Couffin A.-C., Vinet F., Texier-Nogues I. (2011). Lipidots: competitive organic alternative to quantum dots for in vivo fluorescence imaging. J. Biomed. Opt..

[29] Dey A.K., Nougarède A., Clément F., Fournier C., Jouvin-Marche E., Escudé M., Jary D., Navarro F.P., Marche P.N. (2021). Tuning the Immunostimulation properties of cationic lipid Nanocarriers for nucleic acid delivery. Front. Immunol..

[30] Thoo L., Noti M., Krebs P. (2019). Keep calm: the intestinal barrier at the interface of peace and war. Cell Death Dis..

[31] Turner J.R. (2009). Intestinal mucosal barrier function in health and disease. Nat. Rev. Immunol..

[32] Patankar J.V., Becker C. (2020). Cell death in the gut epithelium and implications for chronic inflammation. Nat. Rev. Gastroenterol. Hepatol..

[33] Fasano A. (2012). Leaky gut and autoimmune diseases. Clin. Rev. Allergy Immunol..

[34] Camilleri M., Enteric C., Translational N., Clinic M. (2020). The leaky gut: mechanisms. Measurement and Clinical Implications in Humans.

[35] Liu J.J., Wong K., Thiesen A.L., Mah S.J., Dieleman L.A., Claggett B., Saltzman J.R., Fedorak R.N. (2011). Increased epithelial gaps in the small intestines of patients with inflammatory bowel disease: density matters. Gastrointest. Endosc..

[36] Kiesslich R., Duckworth C.A., Moussata D., Gloeckner A., Lim L.G., Goetz M., Pritchard D.M., Galle P.R., Neurath M.F., Watson A.J.M. (2012). Local barrier dysfunction identified by confocal laser endomicroscopy predicts relapse in inflammatory bowel disease. Gut.

[37] Martini E., Krug S.M., Siegmund B., Neurath M.F., Becker C. (2017). Mend your fences: the epithelial barrier and its relationship with mucosal immunity in inflammatory bowel disease. Cmgh.

[38] Yeste J., Illa X., Alvarez M., Villa R. (2018). Engineering and monitoring cellular barrier models. J. Biol. Eng..

[39] Fleisher D. (1999).

[40] Sambuy Y., De Angelis I., Ranaldi G., Scarino M.L., Stammati A., Zucco F. (2005). The Caco-2 cell line as a model of the intestinal barrier: influence of cell and culture-related factors on Caco-2 cell functional characteristics. Cell Biol. Toxicol..

[41] Li N., Wang D., Sui Z., Qi X., Ji L., Wang X., Yang L. (2013). Development of an improved three-dimensional in vitro intestinal mucosa model for drug absorption evaluation. Tissue Eng. - Part C Methods.

[42] Yi B., Shim K.Y., Ha S.K., Han J., Hoang H.H., Choi I., Park S., Sung J.H. (2017). Three-dimensional in vitro gut model on a villi-shaped collagen scaffold. Biochip J..

[43] Michiba K., Maeda K., Kurimori K., Enomoto T., Shimomura O., Takeuchi T., Nishiyama H., Oda T., Kusuhara H. (2021). Characterization of the human intestinal drug transport with ussing chamber system incorporating freshly isolated human jejunum. Drug Metab. Dispos..

[44] Leonard F., Ali H., Collnot E.M., Crielaard B.J., Lammers T., Storm G., Lehr C.M. (2012). Screening of budesonide nanoformulations for treatment of inflammatory bowel disease in an inflamed 3D cell-culture model. ALTEX.

[45] Kämpfer A.A.M., Urbán P., Gioria S., Kanase N., Stone V., Kinsner-Ovaskainen A. (2017). Development of an in vitro co-culture model to mimic the human intestine in healthy and diseased state. Toxicol. in Vitro.

[46] Spano A., Barni S., Sciola L. (2013). PMA withdrawal in PMA-treated monocytic THP-1 cells and subsequent retinoic acid stimulation, modulate induction of apoptosis and appearance of dendritic cells. Cell Prolif..

[47] Park E.K., Jung H.S., Yang H.I., Yoo M.C., Kim C., Kim K.S. (2007). Optimized THP-1 differentiation is required for the detection of responses to weak stimuli. Inflamm. Res..

[48] Koohestani F., Braundmeier A.G., Mahdian A., Seo J., Bi J.J., Nowak R.A. (2013). Extracellular matrix collagen alters cell proliferation and cell cycle progression of human uterine leiomyoma smooth muscle cells. PLoS One.

[49] Jones G.R., Bain C.C., Fenton T.M., Kelly A., Brown S.L., Ivens A.C., Travis M.A., Cook P.C., MacDonald A.S. (2018). Dynamics of colon monocyte and macrophage activation during colitis. Front. Immunol..

[50] Hart A.L., Al-Hassi H.O., Rigby R.J., Bell S.J., Emmanuel A.V., Knight S.C., Kamm M.A., Stagg A.J. (2005). Characteristics of intestinal dendritic cells in inflammatory bowel diseases. Gastroenterology.

[51] Xiao B., Merlin D. (2012). Oral colon-specific therapeutic approaches toward treatment of inflammatory bowel disease. Expert Opin Drug Deliv..

[52] Ali H., Collnot E.M., Windbergs M., Lehr C.M. (2013). Nanomedicines for the treatment of inflammatory bowel diseases. Eur J. Nanomedicine.

[53] Huckaby J.T., Lai S.K. (2018). PEGylation for enhancing nanoparticle diffusion in mucus. Adv. Drug Deliv. Rev..

[54] Murgia X., Loretz B., Hartwig O., Hittinger M., Lehr C.-M.M. (2018). The role of mucus on drug transport and its potential to affect therapeutic outcomes. Adv. Drug Deliv. Rev..

[55] Salas A., Hernandez-Rocha C., Duijvestein M., Faubion W., McGovern D., Vermeire S., Vetrano S., Vande Casteele N. (2020). JAK–STAT pathway targeting for the treatment of inflammatory bowel disease. Nat. Rev. Gastroenterol. Hepatol..

[56] Danese S., Grisham M., Hodge J., Telliez J.-B. (2016). JAK inhibition using tofacitinib for inflammatory bowel disease treatment: a hub for multiple inflammatory cytokines. Am. J. Physiol. Gastrointest. Liver Physiol..

[57] Beattie D.T., Pulido-Rios M.T., Shen F., Ho M., Situ E., Tsuruda P.R., Brassil P., Kleinschek M., Hegde S. (2017). Intestinally-restricted Janus kinase inhibition: a potential approach to maximize the therapeutic index in inflammatory bowel disease therapy. J. Inflamm. (United Kingdom).

[58] Krutzik P.O., Nolan G.P. (2003). Intracellular phospho-protein staining techniques for flow cytometry: monitoring single cell signaling events. Cytom. Part A.

[59] Cohen S., Dovrat S., Sarid R., Huberman E., Salzberg S. (2005). JAK–STAT signaling involved in phorbol 12-myristate 13-acetate- and dimethyl sulfoxide-induced 2′-5′ oligoadenylate synthetase expression in human HL-60 leukemia cells. Leuk. Res..

[60] Ivashkiv L.B. (2018). IFNγ: signalling, epigenetics and roles in immunity, metabolism, disease and cancer immunotherapy. Nat. Rev. Immunol..

[61] Virtanen T., Haikarainen T., Raivola J., Silvennoinen O. (2019). Selective JAKinibs: prospects in inflammatory and autoimmune diseases. BioDrugs.

[62] Hodge J.A., Kawabata T.T., Krishnaswami S., Clark J.D., Telliez J.B., Dowty M.E., Menon S., Lamba M., Zwillich S. (2016). The mechanism of action of tofacitinib - an oral Janus kinase inhibitor for the treatment of rheumatoid arthritis. Clin. Exp. Rheumatol..

[63] Sandborn W.J., Nguyen D.D., Beattie D.T., Brassil P., Krey W., Woo J., Situ E., Sana R., Sandvik E., Pulido-Rios M.T., Bhandari R., Leighton J.A., Ganeshappa R., Boyle D.L., Abhyankar B., Kleinschek M.A., Graham R.A., Panes J. (2020). Development of gut-selective pan-janus kinase inhibitor TD-1473 for ulcerative colitis: a translational medicine programme. J. Crohn’s Colitis.

[64] Rybakovsky E., Valenzano M.C., Diguilio K.M., Buleza N.B., Moskalenko D.V., Harty R.N., Mullin J.M. (2019). Improving transient transfection efficiency in a differentiated, polar epithelial cell layer. J. Biomol. Tech..

