# Supplementary material for: Leaky gut model of the human intestinal mucosa for testing siRNA-based nanomedicine targeting JAK1
Source: J Control Release. 2022 May;345:646–60. doi: 10.1016/j.jconrel.2022.03.037 (PMC9168449; doi:10.1016/j.jconrel.2022.03.037)
Supplement: Supplementary file 1 — Supplementary material [file mmc1.pdf]

Leaky gut model of the human intestinal mucosa for testing siRNA-based nanomedicine targeting JAK1

Olga Hartwig[1,2], Brigitta Loretz[1], Adrien Nougarede[3,4], Dorothée Jary[3,4], Eric Sulpice[5], Xavier Gidrol[5], Fabrice Navarro[3,4] and Claus-Michael Lehr[1,2]

[1] Helmholtz Institute for Pharmaceutical Research Saarland (HIPS) - Helmholtz Centre for Infection Research (HZI), D-66123 Saarbrücken, Germany
[2] Department of Pharmacy, Saarland University, D-66123 Saarbrücken, Germany
[3] University Grenoble Alpes, F-38000 Grenoble, France
[4] CEA LETI, Minatec Campus, F-38054, Grenoble, France
[5] University Grenoble Alpes, CEA, INSERM, IRIG, Biomics, F-38000 Grenoble, France

E-mail: Brigitta.Loretz@helmholtz-hips.de

Supporting information (SI)

SI Figure 1 Surface area measurements for Caco-2 confluence calculation.

SI Figure 2 Representative graphs obtained from automated CASY Cell counter with cell type-specific size distributions.

SI Figure 3 Live/dead cell staining of cell suspension after dissociation of tight barrier (A) and leaky gut (B) model using flow cytometry.

SI Figure 4 Confocal microscopic images of tight barrier model after nanoplex incubation using fluorescently-labeled nanocarrier (DiI-labeled LNPs) and siRNA (AF647-labeled siJAK1).

SI Figure 5 Confocal microscopic images of leaky gut model after nanoplex incubation using fluorescently-labeled nanocarrier (DiI-labeled LNPs) and siRNA (AF647-labeled siJAK1).

SI Figure 6 Flow cytometric analysis of nanoplex association in leaky gut model.

SI Figure 7 Flow cytometric analysis of STAT1 phosphorylation (pY701) using anti-phospho-STAT1 antibodies and assay protocol adaptation for JAK/STAT pathway activation and downregulation.

SI Figure 8 Confocal microscopic images of leaky gut model and cell morphology of sub-epithelial immune cells.

SI Figure 9 Macrophage-like phenotype of dTHP-1 after PMA-differentiation and expression of different cell markers determined by flow cytometry and confocal microscopy.

SI Figure 10 Confocal microscopic images of co-culture in absence of Caco-2 epithelial cell layer.

SI Figure 11 Confocal microscopic images of tight barrier model comprising a confluent Caco-2 cell layer on top and uptake study of LNPs.

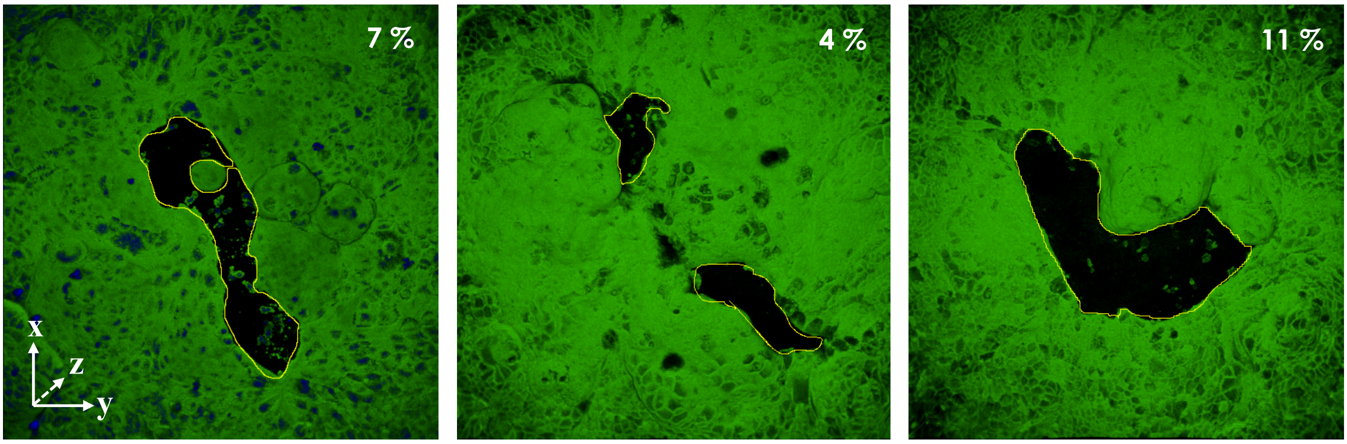

SI Figure 1 Surface area measurements for Caco-2 confluence calculation. Examples of confocal images with sub-confluent regions within the leaky gut model in top view (xy-dimension) and overlaid fluorescence channels. Co-culture was counterstained for cell nuclei (DAPI; blue) and cytoskeleton (AF488 phalloidin; green). Caco-2 confluency of the leaky gut model was calculated after 6/7 days of cultivation by ImageJ (version 1.52, NIH, Bethesda, USA). After ´free hand´ selection of Caco-2 cell-free areas (yellow line), respective regions were measured using ´Analyze > Measure´ and the resulting area divided by the total area of the image (represented as percentage of confluency).

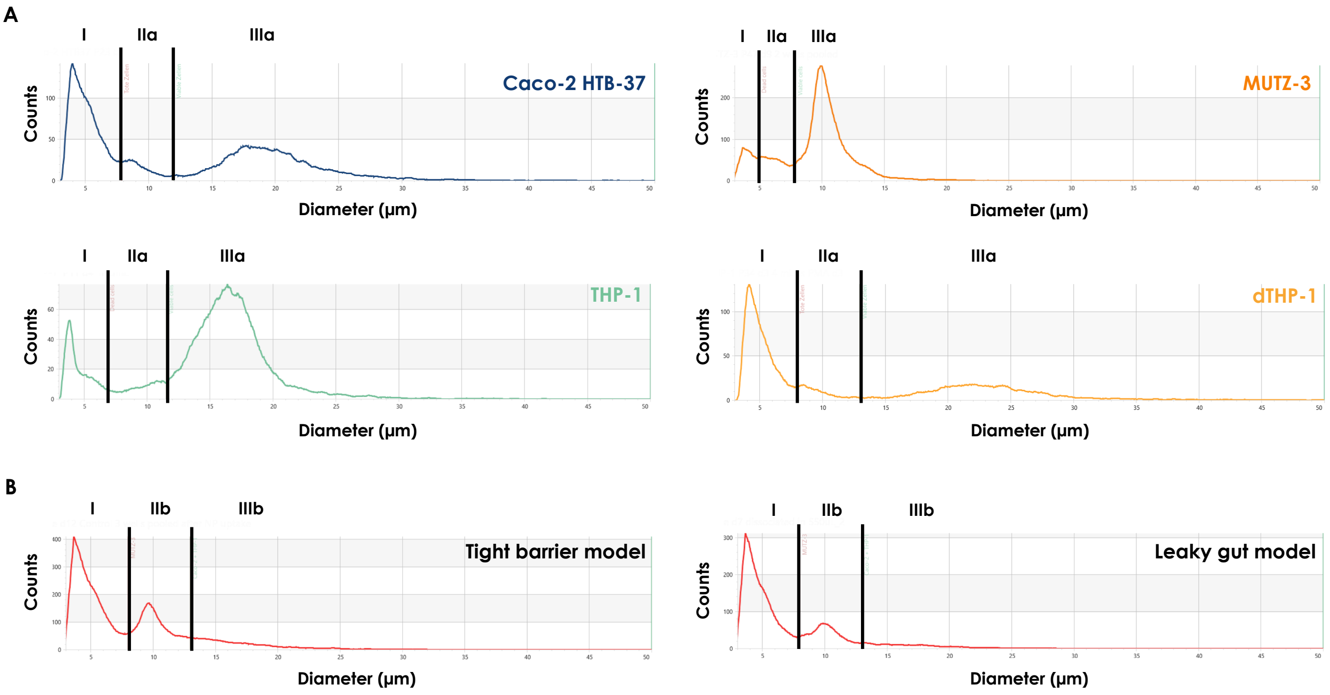

SI Figure 2 Representative graphs obtained from automated CASY Cell counter with cell type-specific size distributions. Representative graphs of cell size distribution for Caco-2, MUTZ-3, THP-1, and dTHP-1 cells (**A**). Cell number and percentage viability was determined by different size ranges pre-set for each individual cell line in order to differentiate between cell debris (< 8 µm; **I**), dead or dying cells (**IIa**), and viable cell population (**IIIa**). Representative graphs of resulting single-cell suspension after dissociation of tight barrier and leaky gut model (**B**). Appropriate size ranges were determined to separate for dendritic-like cells (MUTZ-3) with a range of 8-13 µm (**IIb**) and the other two cell types, epithelial (Caco-2) and macrophage-like cells (dTHP-1), with similar size ranges between 13-50 µm (**IIIb**).

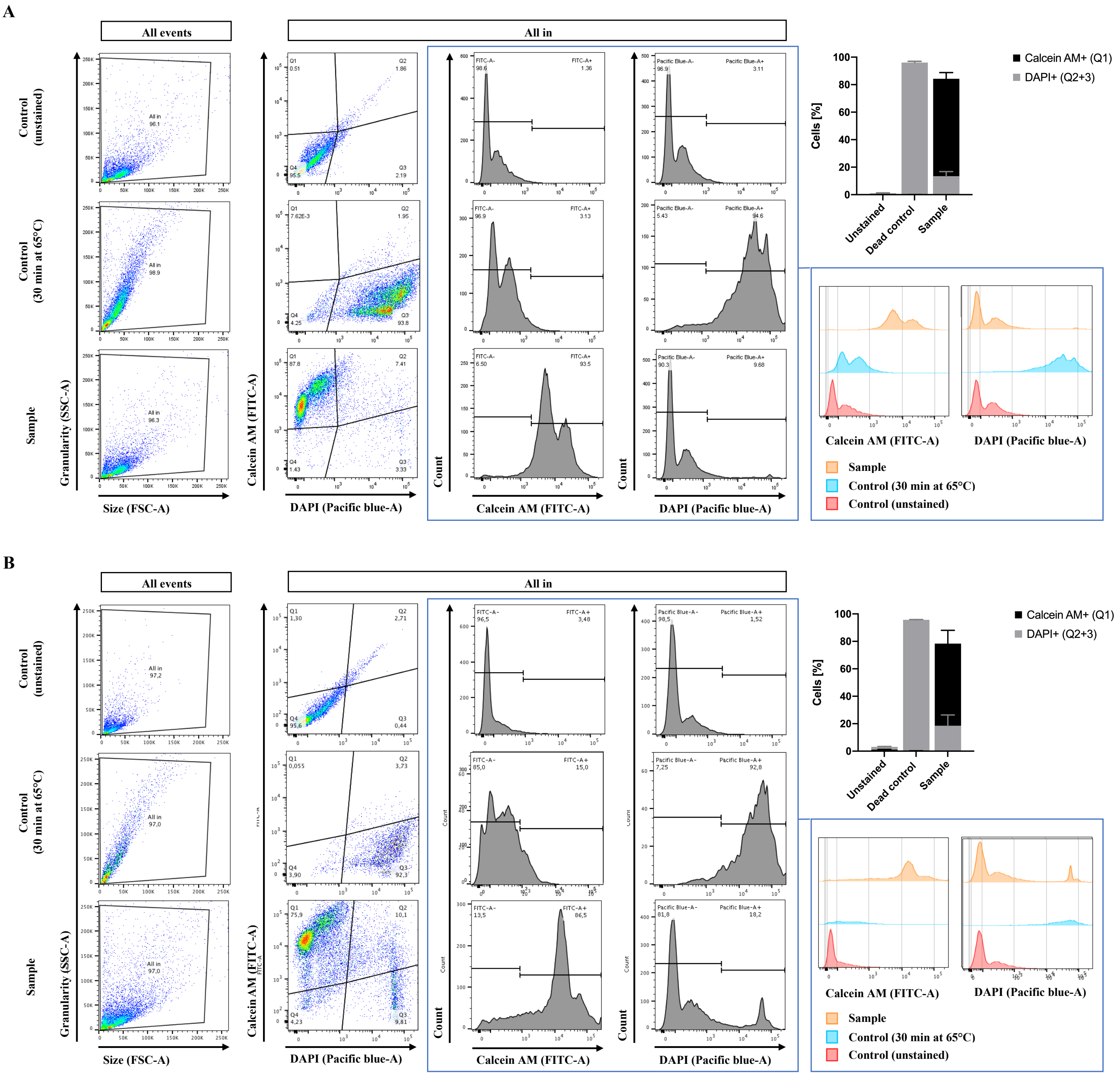

SI Figure 3 Live/dead cell staining of cell suspension after dissociation of tight barrier (A) and leaky gut (B) model using flow cytometry. Resulting single cells suspension was stained for living (Calcein AM; 10 ng/mL) and dying/dead cells (DAPI; 0.1 µg/mL) and analyzed by FACS (10.000 counts). Representative flow cytometric data with gating strategy to identify viable (Calcein AM- or FITC-positive) and dead cell fraction (DAPI- or Pacific blue-positive). Obtained cell populations are visualized in granularity *vs*. size (SSC-A/FSC-A) and fluorescence (FITC-A/Pacific blue-A) density plots or histograms with separated fluorescence channels with positive-cell fractions. Upper panel shows control of unstained cells (without addition of Calcein AM/DAPI) and a double-negative cell population (dot plot; Q4). The middle panel displays the stained dead cell control (heat-treated at 65°C for 30 min) with Calcein AM-negative and DAPI-positive cells. Lower panel shows the stained cell suspension with distinct Calcein AM- and DAPI-positive cell fractions (Sample). Overlaid histograms are highlighted in the blue box. Calcein AM- and DAPI-positive cell population are summarized as percentages of positive cells (mean ± SD).

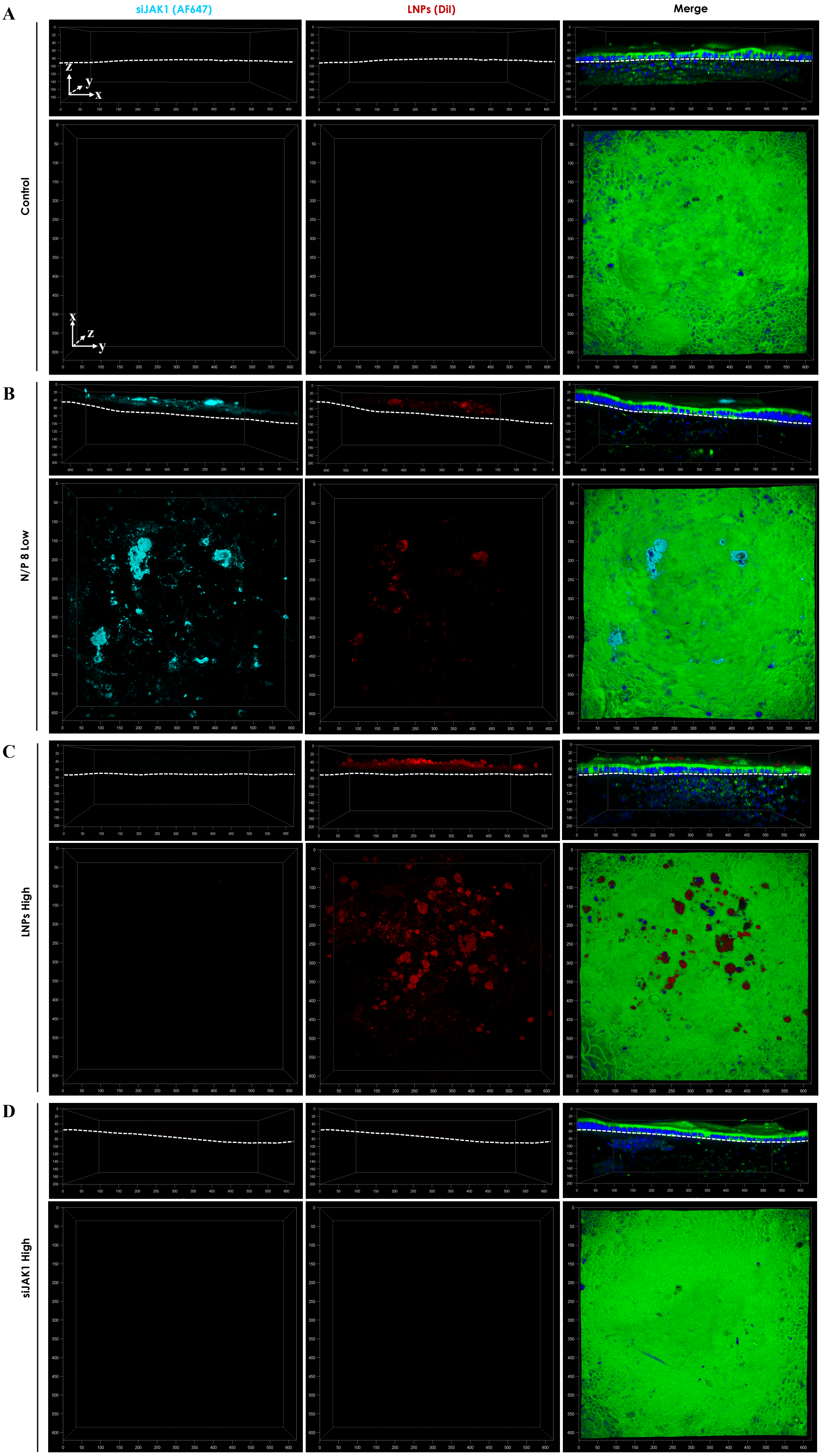
A
siJAK1 (AF647)
LNPs (DiI)
Merge
Control
B
N/P 8 Low
C
LNPs High
D
siJAK1 High

SI Figure 4 Confocal microscopic images of tight barrier model after nanoplex incubation using fluorescently-labeled nanocarrier (DiI-labeled LNPs) and siRNA (AF647-labeled siJAK1). Tight barrier model in absence of nanoplexes (control) was used to set up the optimal fluorescence detection of respective fluorophores of the nanocarrier (DiI-LNPs) and cargo (AF647-siJAK1) without generating unspecific background signal (**A**). Nanoplexes with N/P ratio of 8 (45 µg LNPs and 135 nM siJAK1; N/P 8 Low) (**B**), and control treatments were incubated twice (apically) for 6 h including blank LNPs (90 µg per well; LNPs High) (**C**), and naked siJAK1 (270 nM; siJAK1 High) (**D**). Afterwards, cells were fixed and stained for cytoskeleton (AF488-phalloidin; green) and cell nuclei (DAPI; blue). Recorded z-stacks of around 200 µm depth (axis unit = µm) are represented in top (xy-) or side (xz/yz-dimension) view with overlaid fluorescence signal (merge) or split channels for DiI-LNPs (red) and AF647-siJAK1 (cyan). Basal site of epithelial cell layer (attached to the collagen matrix) is marked by a dashed line to identify the localization of nanocarrier and/or its cargo.

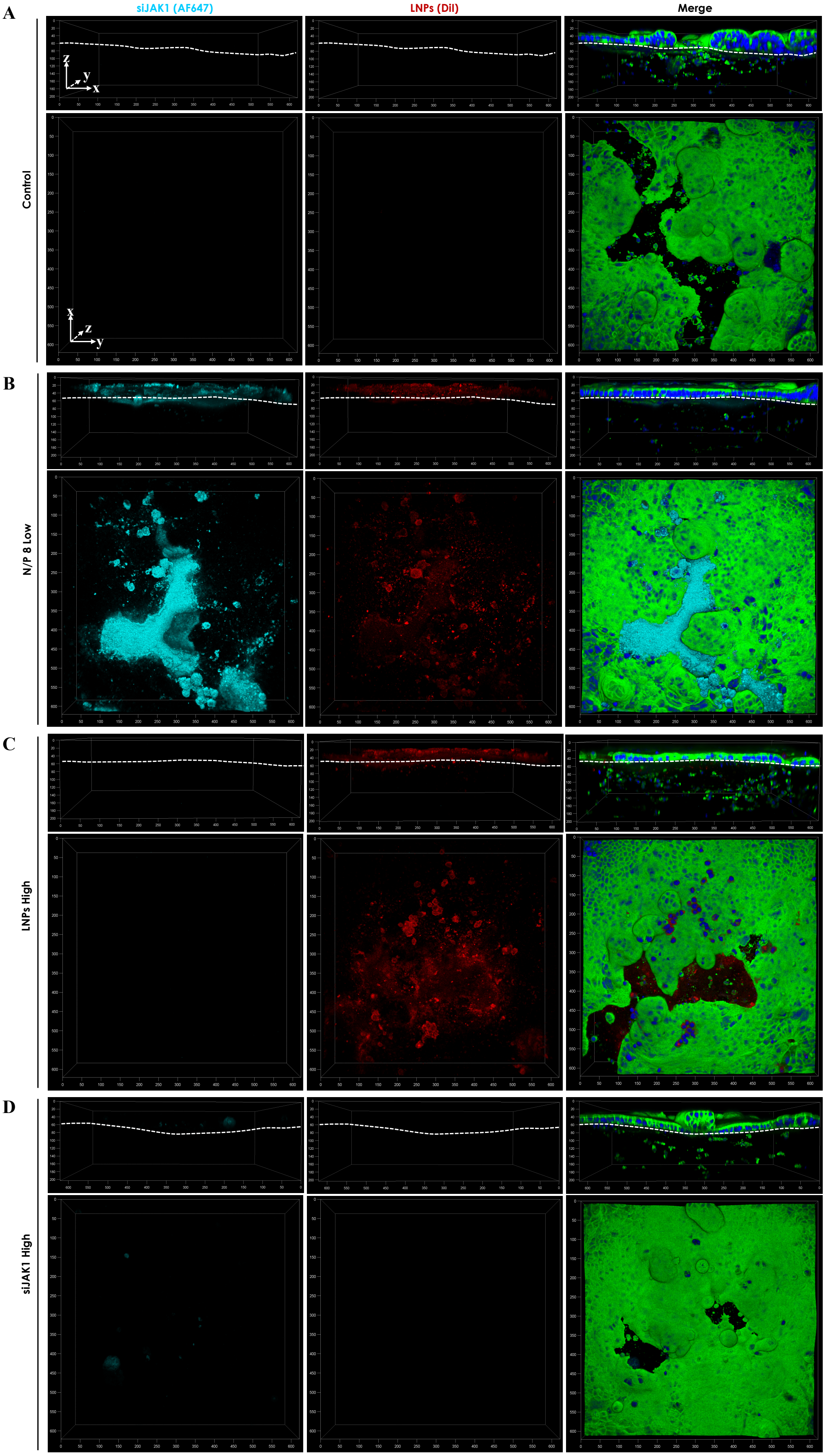
A
B
C
D
siJAK1 (AF647)
LNPs (DiI)
Merge
Control
N/P 8 Low
LNPs High
siJAK1 High

SI Figure 5 Confocal microscopic images of leaky gut model after nanoplex incubation using fluorescently-labeled nanocarrier (DiI-labeled LNPs) and siRNA (AF647-labeled siJAK1). Leaky gut model in absence of nanoplexes (control) was used to set up the optimal fluorescence detection of respective fluorophores of nanocarrier (DiI-LNPs) and cargo (AF647-siJAK1) without generating unspecific background signal (**A**). Nanoplexes with N/P ratio of 8 (45 µg LNPs and 135 nM siJAK1; N/P 8 Low) (**B**), and control treatments were incubated twice (apically) for 6 h including blank LNPs (90 µg per well; LNPs High) (**C**), and naked siJAK1 (270 nM; siJAK1 High) (**D**). Afterwards, cells were fixed and stained for cytoskeleton (AF488-phalloidin; green) and cell nuclei (DAPI; blue). Recorded z-stacks of around 200 µm depth (axis unit = µm) are represented in side (xz/yz-dimension; upper panel) or top (xy-dimension; lower panel) view with overlaid fluorescence signal (merge) or split channels for DiI-LNPs (red) and AF647-siJAK1 (cyan). Basal site of epithelial cell layer (attached to the collagen matrix) is marked by a dashed line to identify the localization of nanocarrier and/or its cargo.

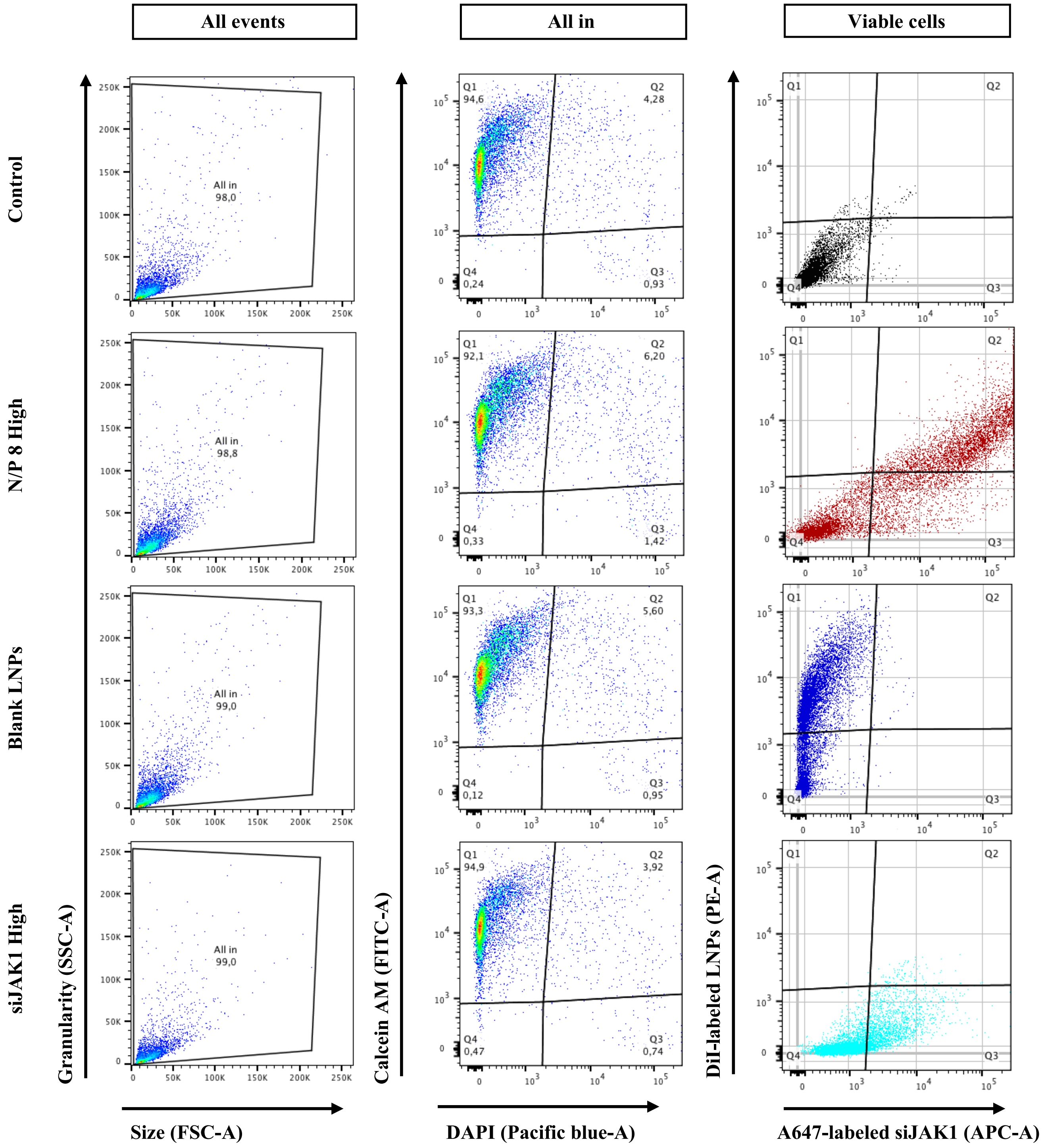

SI Figure 6 Flow cytometric analysis of nanoplex association in leaky gut model. Leaky gut model was treated twice for 6 h with fluorescently-labeled nanoplexes at N/P ratio of 8 (90 µg LNPs and 270 nM siJAK1; N/P 8 High). Control treatments were co-cultures without nanocarrier and siRNA (Control), blank LNPs (90 µg/mL) or naked siJAK1 (270 nM; High). Afterwards, leaky gut model was dissociated and resulting cell suspension stained for living (Calcein AM; 10 ng/mL) and dying/dead cells (DAPI; 0.1 µg/mL). Cells are represented in granularity *vs.* size (SSC-A/FSC-A; left column) and fluorescence density plots identifying viable cells (FITC-A/Pacific blue-A, middle column) and nanoplex association within the viable cell population (PE-A/APC-A; right column).

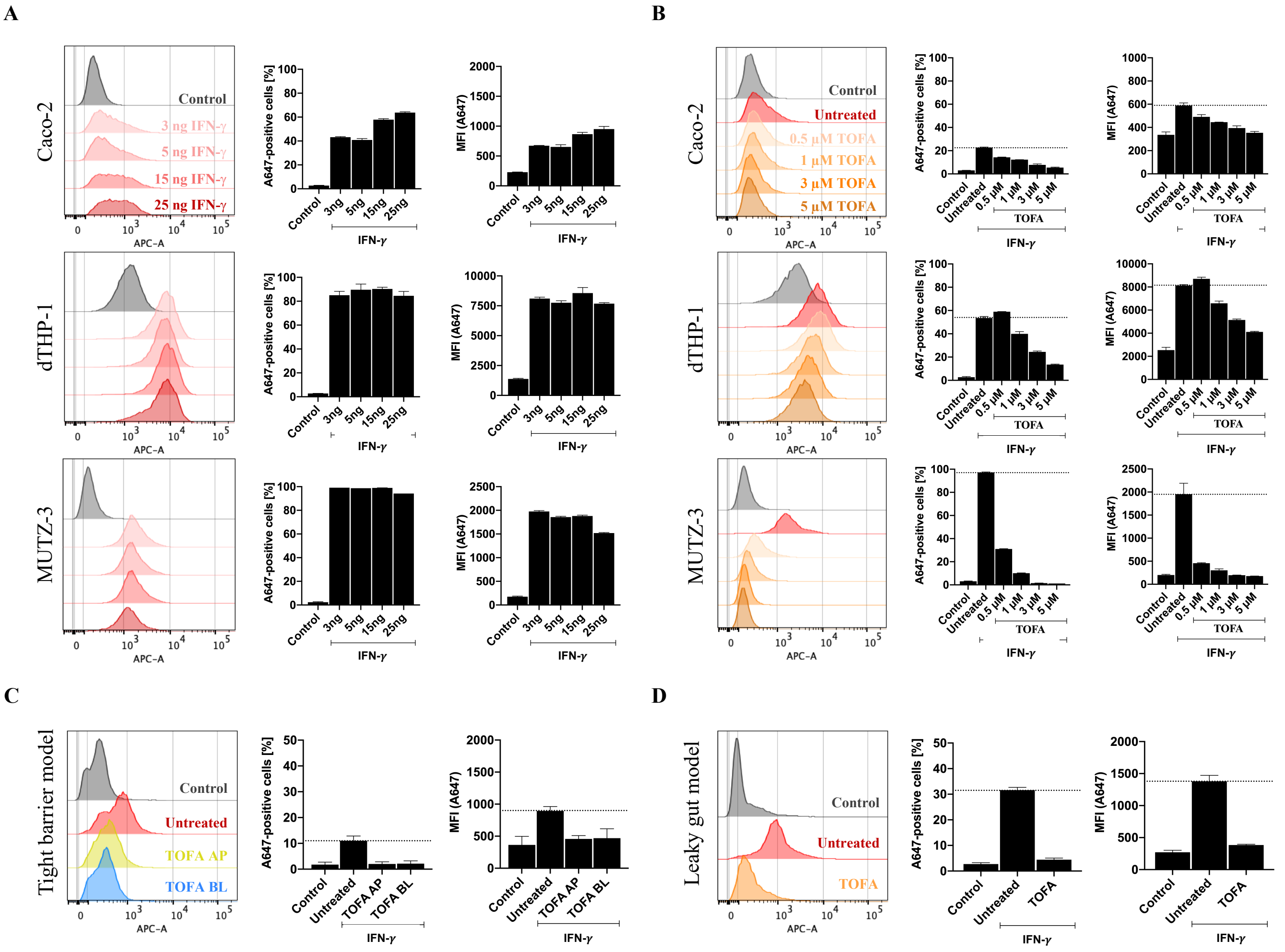

SI Figure 7 Flow cytometric analysis of STAT1 phosphorylation (pY701) using anti-phospho-STAT1 antibodies and assay protocol adaptation for JAK/STAT pathway activation and downregulation. STAT1 activation was tested in different mono-cultures (Caco-2, dTHP-1 and MUTZ-3) stimulated with IFN-γ for 1 h ranging from 3 to 25 ng/well (**A**). Cells were pre-treated twice with TOFA (0.5 to 5 µM) for 6 h on two subsequent days and afterwards stimulated with 25 ng/well IFN-γ (50 ng/mL) for 1 h (**B**). Phosphorylation profile of pSTAT1 of leaky gut model pre-treated twice on day 6/7 with 5 µM TOFA for 6 h applied to both compartments (**C**) and of tight barrier model pre-treated on day 11/12 with 5 µM TOFA for 6 h applied either to the apical (AP) or basolateral (BL) compartment (**D**). After pre-treatment, both co-cultures were stimulated with 50 ng/mL IFN-γ for 1 h and subsequently dissociated for phospho-specific STAT1 staining and FACS analysis. Phosphorylation of STAT1 is represented as histogram overlay with AF647 emission collected at APC instrument setting (APC-A) and summarized in both percentage of pSTAT1-positive cells (%) and mean fluorescence intensity (MFI). pSTAT1-positive cells are compared to unstimulated (control; grey), untreated cells (red), and TOFA pre-treatments applied to the apical (AP; yellow), basolateral (BL; violet), or both compartments (orange). Data of are means ± SD, for mono-cultures (n=3; N=1), tight model (n=9; N=3), and leaky model (n=15; N=5).

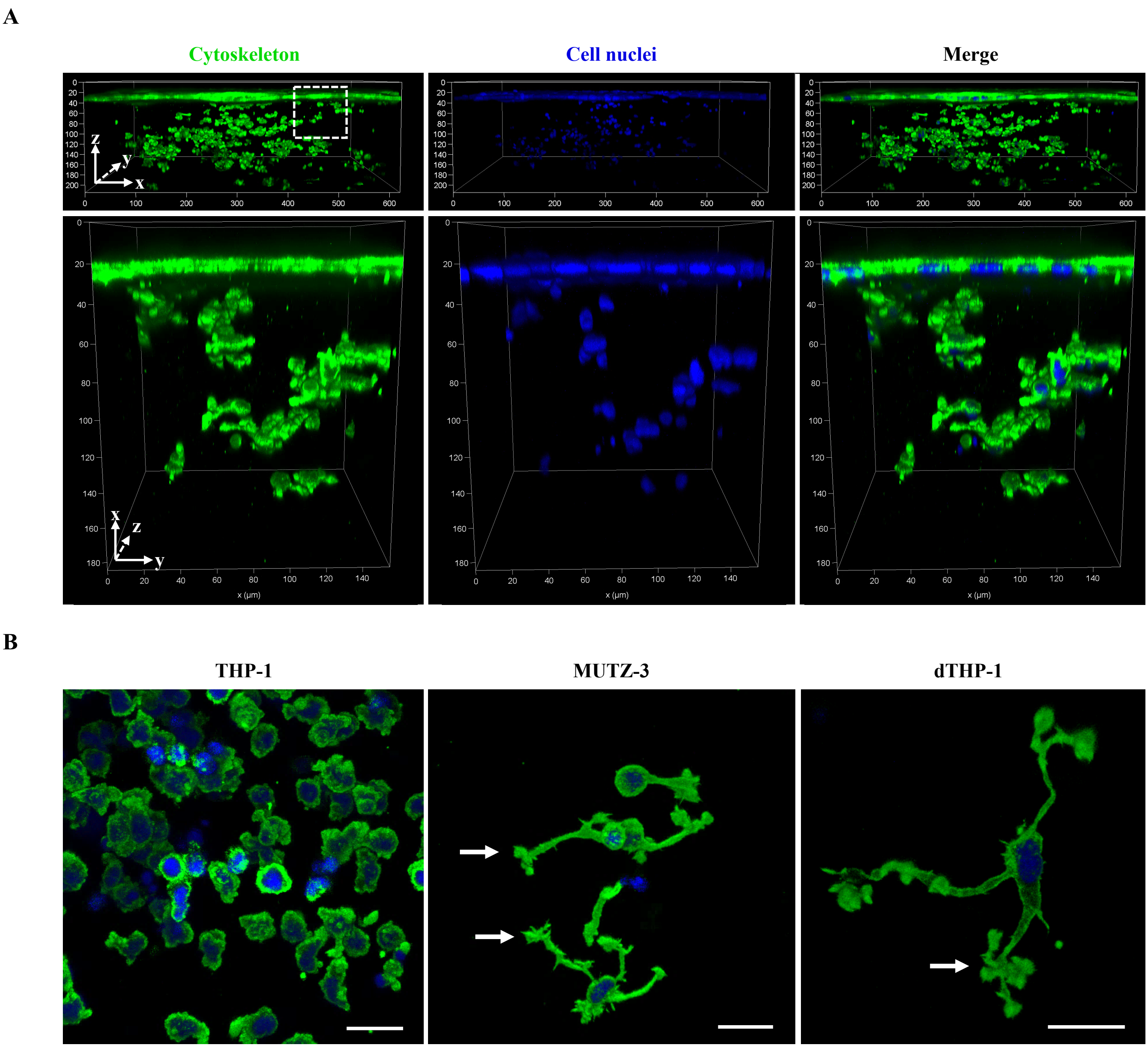

SI Figure 8 Confocal microscopic images of leaky gut model and cell morphology of sub-epithelial immune cells. Leaky gut model with closeup of immune cells in close proximity to epithelial cell layer (**A**). Immune cell morphology with round shaped THP-1 cells and cell protrusions with podosomal-like structures (white arrows) of MUTZ-3/dTHP-1 cells (**B**). Images of 3D cells are projections of 20-30 µm z-sections. (**B**). Cells were stained for cytoskeleton (AF488-phalloidin; green) and cell nuclei (DAPI; blue). Recorded z-stacks of around 200 µm depth are represented in top (xy-) or side (xz/yz-dimension) view with overlaid (merged) fluorescence signal. (axis unit = µm; scale bar = 20 µm).

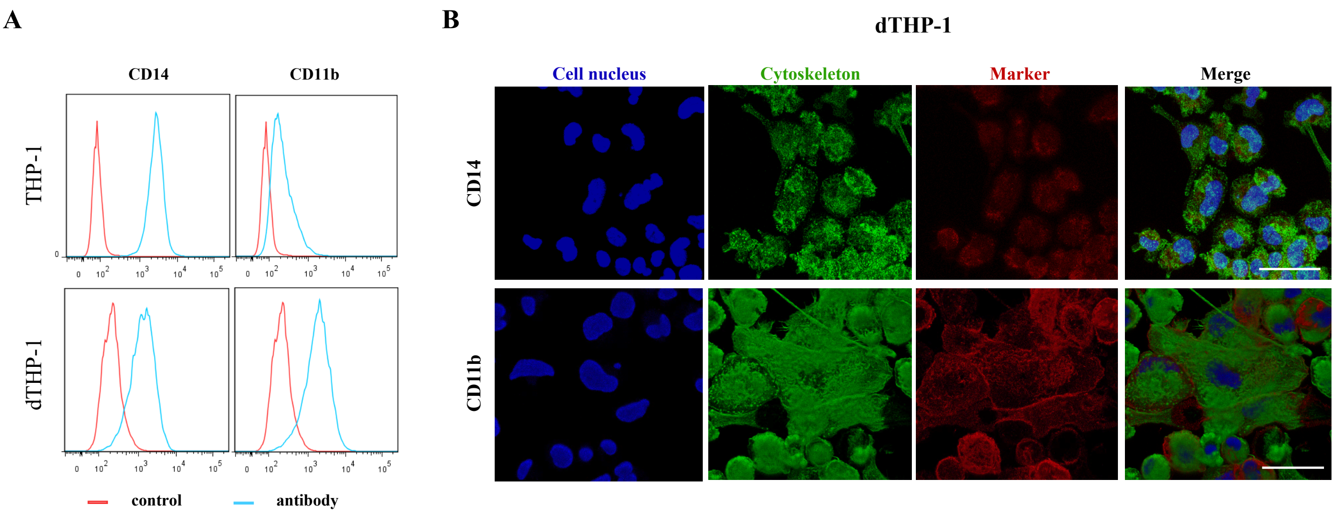

SI Figure 9 Macrophage-like phenotype of dTHP-1 after PMA-differentiation and expression of different cell markers determined by flow cytometry and confocal microscopy. CD11b (β2 integrin; macrophage marker) and CD14 (monocyte marker) was estimated by flow cytometry in monocytic (THP-1) and PMA-differentiated (dTHP-1) cells (**A**). THP-1 cells were seeded with or without PMA (50 nM; 72 hours) and harvested for flow cytometric analysis. Histogram overay displays FITC-positive (antibody) cells compared to unstained (control). Confocal microscopic images of dTHP-1 cells (seeded on µslides) stained with mouse anti-CD14 (FITC) or anti-CD11b (AF647) antibodies (**B**). Cells were stained for cytoskeleton (Rhodamine or AF488-phalloidin; green) and cell nuclei (DAPI; blue). Recorded images are represented in split channels or overlaid (merged) fluorescence signal with both cell markers shown in red (Scale bar = 50 µm).

A

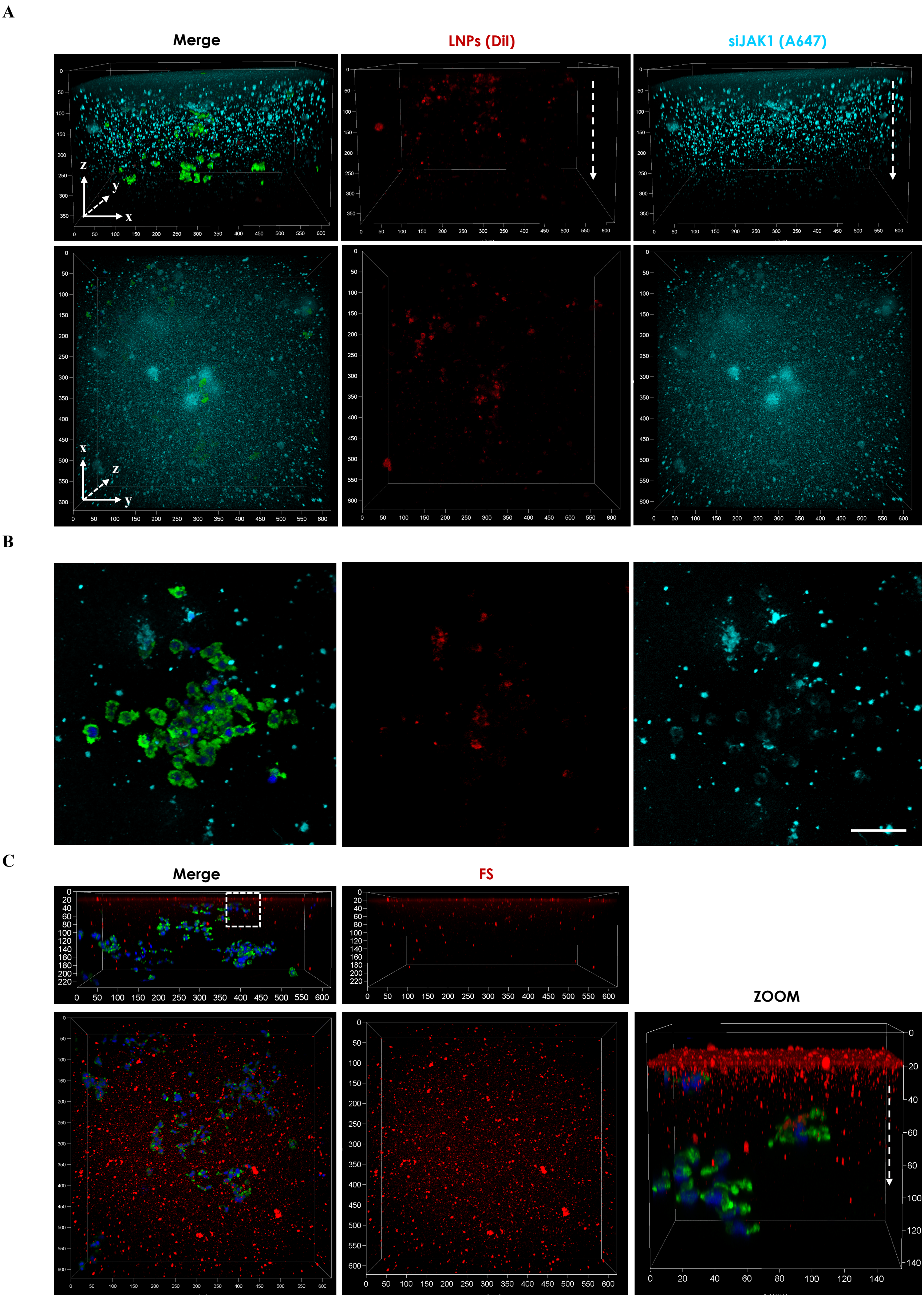

SI Figure 10 Confocal microscopic images of co-culture in absence of Caco-2 epithelial cell layer. Co-culture model without Caco-2 cells on top was used to verify the penetration of nanocarrier through the collagen matrix. Fluorescently-labeled nanocarrier (DiI-labeled LNPs; 90 µg/well) complexed with siRNA (AF647-siJAK1; 450 nM) were applied apically and incubated for 6 hours (**A**). Uptake of nanocarriers by immune cells within the collagen matrix (top view). Images of 3D cells are projections of 20-30 µm z-sections (scale bar = 50µm). (**B**). In comparison, diffusion of carboxylated polystyrene nanoparticles (FluoSpheres®; FS) of 100 nm size (50 µg/well) after 6 h of incubation (**C**). Cells are stained for cytoskeleton (AF488-phalloidin; green) and cell nuclei (DAPI; blue). Recorded z-stacks of around 350 µm depth (axis unit = µm) are represented in top (xy-) or side (xz/yz-dimension) view with overlaid (merged) fluorescence signal or in split channels for DiI-LNPs and FS (red) and AF647-siJAK1 (cyan).

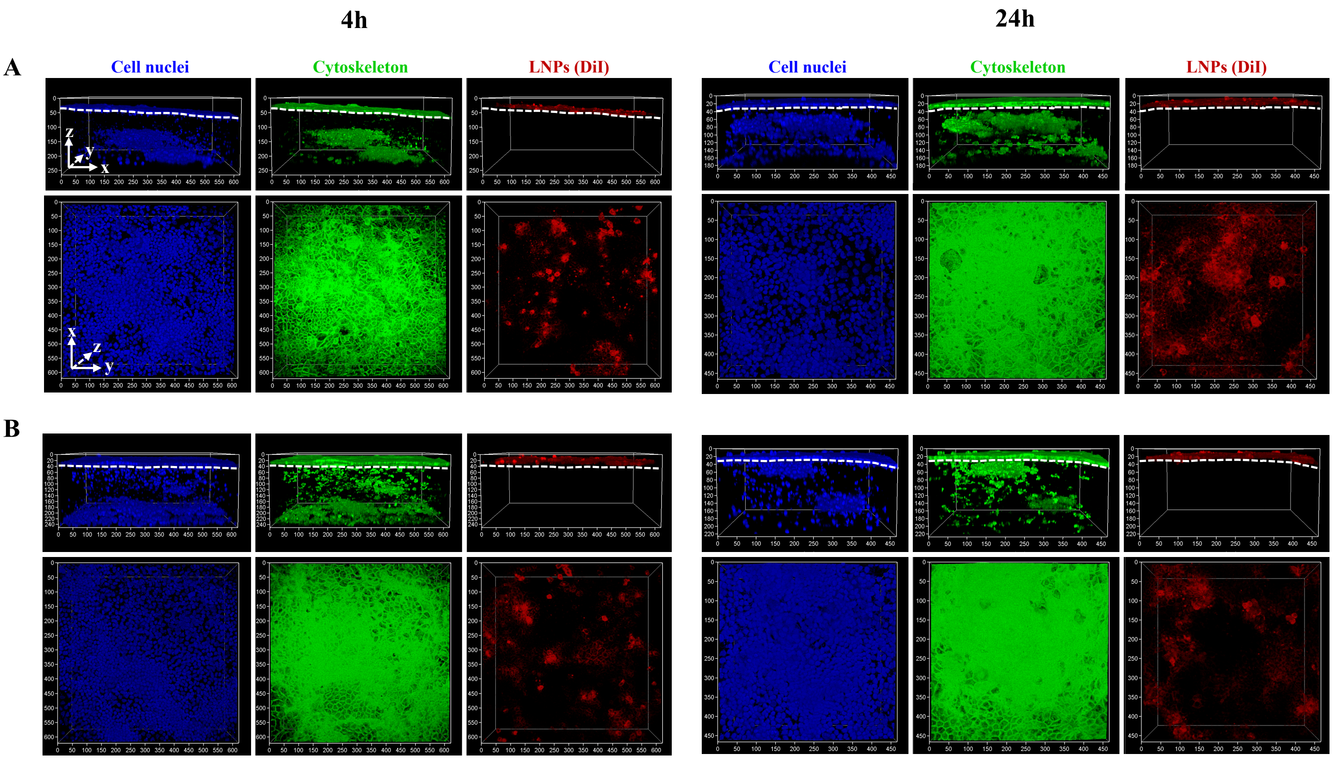

SI Figure 11 Confocal microscopic images of tight barrier model comprising a confluent Caco-2 cell layer on top and uptake study of LNPs. Nanocarriers were applied apically (90 µg/well) and incubated for either 4 or 24 h in a healthy (**A**) and LPS-inflamed co-culture model (**B**). For all conditions, co-localization of nanocarriers within epithelial cells and barrier function decrease upon LPS-inflammation do not increase particle penetration and access to the underlying immune cells, even for longer incubation time. Recorded z-stacks of around 250 µm depth (axis unit = µm) are represented in top (xy-) or side (xz/yz-dimension) view in split fluorescence channels for cytoskeleton (AF488-phalloidin; green), cell nuclei (DAPI; blue), and nanocarrier (DiI-labeled LNPs; red). Basal site of epithelial cell layer (attached to the collagen matrix) is marked by a dashed line to identify the localization of nanocarrier.
